# Evaluating longitudinal speed control dynamics approaching work zones: the impact of visual–auditory interventions

**DOI:** 10.3389/fpsyg.2026.1961865

**Published:** 2026-10-01

**Authors:** Jie Li, Yadikaer Wumai’er, Bingshuo Chen, Gaoqiang Zhang, Linwei Wang, Dehai Liu, Jinglin Li

**Affiliations:** 1 Xinjiang Transportation Investment (Group) Co., Ltd., Urumqi, China; 2 Xinjiang Jiaotou Construction Management Co., Ltd., Urumqi, China; 3 Research Institute of Highway, Ministry of Transport, Beijing, China; 4 College of Metropolitan Transportation, Beijing University of Technology, Beijing, China

**Keywords:** driving dynamics, highway work zone, linear mixed-effects model, longitudinal speed control, multimodal warnings

## Abstract

**Introduction:**

Current safety evaluations of highway work zones often focus on cross-sectional speed compliance, overlooking continuous deceleration dynamics. This study evaluates how multimodal warnings combining visual markings and auditory alerts affect spatial deceleration behaviors.

**Methods:**

Using a driving simulator, continuous vehicle trajectories were analyzed using a linear mixed-effects model with spatial interaction terms to account for individual driving habits and examine the effects of different warning interventions.

**Results:**

The results show that physical barricades and mandatory speed limits largely determine maximum deceleration and absolute speed reduction, regardless of warning type. However, multimodal warnings significantly smooth the microscopic deceleration process. By extending reaction times, they reduce panic hard braking and lower peak negative jerk. Furthermore, while isolated auditory alerts are subject to driver adaptation in far-field zones, multimodal combinations effectively counteract driver laxity in high-workload bottleneck segments, reducing the trajectory speeding exposure rate to 39.9%.

**Discussion:**

These findings indicate that multimodal warnings enhance safety primarily by smoothing longitudinal driving trajectories rather than altering absolute braking limits, providing quantitative evidence for spatially differentiated warning layouts.

## 1 Introduction

Highway work zones are widely recognized as high-risk areas within the road network due to physical lane constrictions, traffic reorganization, and frequent speed limit transitions. The upstream transition zone, in particular, is highly susceptible to severe rear-end conflicts triggered by high speed dispersion and mandatory merging behaviors (Weng and Meng, 2014). Empirical studies based on naturalistic driving data further indicate that the complex and dense work zone environment significantly increases drivers’ visual and cognitive workload, leading to a sharp decline in longitudinal control stability (Ma S. et al., 2023). Concurrently, deceleration lag, car-following oscillations, and localized spatiotemporal speeding prevalent after long-distance cruising exponentially amplify the microscopic dynamic conflict risks within the traffic flow (More et al., 2025). Therefore, exploring how external interventions can effectively induce drivers to establish a stable and safe deceleration transition behavior in the upstream approach phases has become a core issue in proactive traffic safety research.

Current active speed control strategies in work zones rely primarily on physical visual interventions, such as transverse deceleration markings, optical speed bars, and roadside visual guidance devices. The core mechanism of these countermeasures lies in restructuring the optical flow field and edge rate within the driver’s field of view. This cognitive intervention induces a perceptual illusion of speed acceleration and spatial distance compression, thereby prompting proactive deceleration intents (Charlton, 2007). For instance, the progressive compression of transverse marking spacing can significantly amplify drivers’ subjective perception of speeding, thus eliciting more active longitudinal braking maneuvers (Godley et al., 2000). Furthermore, in hazardous alignment bottlenecks (e.g., sharp curves), chevron markings and enhanced delineators have been proven effective in optimizing drivers’ visual attention allocation and improving lateral trajectory retention capabilities (Ćelić et al., 2025). Previous high-fidelity driving simulator studies have also confirmed that continuous visual deceleration facilities can effectively reduce global operating speeds and partially enhance car-following stability within work zone environments (Ding et al., 2013).

However, warning systems relying exclusively on the visual channel exhibit inherent limitations in complex traffic environments. The driving task itself already heavily occupies drivers’ visual–spatial cognitive resources (Wickens, 2008), while the high-frequency speed limit recognition, path reconstruction, and complex traffic flow interactions within work zones further lead to a severe overload of visual workload (Yang et al., 2023). Particularly during long-distance cruising or in familiar road environments, drivers are highly susceptible to slipping into an “automated driving” state characterized by reduced situational awareness. Consequently, their peripheral visual scanning breadth and effective fixation frequency on roadside warning devices decay significantly (Charlton and Starkey, 2011). This classic “look-but-fail-to-see” phenomenon indicates that even if drivers execute physical visual scanning, they fail to successfully translate these cues into substantial hazard perception at the cognitive level (Martens and Fox, 2007). Previous eye-tracking studies have also confirmed that drivers familiar with the route exhibit significantly shorter fixation durations on peripheral environmental information compared to unfamiliar drivers (Hu et al., 2022), making them highly prone to missing critical dynamic speed limits and barricade constraints at the forefront of sudden, high-risk work zones.

These limitations can be further interpreted through two complementary theoretical perspectives. According to Multiple Resource Theory, visual and auditory information can draw on partially distinct processing resources, suggesting that the addition of auditory cues may reduce competition within the already heavily loaded visual channel (Wickens, 2008). Meanwhile, Risk Homeostasis Theory suggests that drivers continuously regulate their behavior according to their perceived level of risk, such that external warnings may induce compensatory adjustments in driving behavior without necessarily producing a persistent change in the final speed-control outcome (Wilde, 1982). Together, these theories provide a conceptual basis for examining not only whether multimodal warnings reduce speed, but also how they reshape the continuous deceleration process.

To overcome the information processing bottleneck of the single visual channel, multimodal visual–auditory coordinated warnings have emerged as a frontier direction in proactive intelligent traffic safety research. Auditory stimuli can effectively bypass the highly congested visual processing pathway, instantly awakening drivers’ hazard perception through the cross-modal attention mechanism (Ho et al., 2005). Auditory signals with spatial directional features not only significantly enhance the efficiency of attention capture toward high-risk zones but also substantially compress the cognitive reaction time prior to braking (Ho and Spence, 2005). Recent studies in automated-driving transitions provide further evidence for the benefits of spatially informative auditory and cross-modal warnings. Directional auditory cues and audiovisual coordination have been found to improve attentional guidance and influence takeover-related visual and behavioral responses during automated-driving transitions (Arabian et al., 2026; Ma J. et al., 2023). Although these studies were conducted in automated-driving contexts rather than highway work zones, they provide relevant evidence that directional auditory information can complement visual cues in time-critical driving tasks. Compared to traditional monotonous buzzer alarms, warning modalities with explicit semantic instructions (e.g., directional voice alerts) are more likely to elicit drivers’ accurate instinctive avoidance responses (Belz et al., 1999). Recent meta-analyses further demonstrate that composite warnings incorporating the auditory dimension comprehensively outperform purely visual guidance in response efficiency, remarkably reducing drivers’ braking reaction delays (Zhu et al., 2025). Specifically within highway work zone scenarios, directional auditory interventions have been proven to effectively compensate for visual blind spots in complex traffic environments, fortifying drivers’ recognition acuity of upcoming barricades, and thereby fundamentally suppressing the risk of rear-end collisions in the upstream transition zones (Hang et al., 2022).

Although auditory interventions can enhance drivers’ instantaneous response efficiency, existing studies have primarily focused on discrete macroscopic indicators such as braking reaction time and cross-sectional speed, paying insufficient attention to microscopic longitudinal smoothness during the deceleration process. In fact, abrupt auditory stimuli can easily trigger drivers’ startle responses, leading to unstable maneuvering behaviors such as panic hard braking (Belz et al., 1999). Studies have shown that while startle-based intense alarms shorten hazard response times, they significantly exacerbate the severe oscillations of vehicle longitudinal deceleration (Griffith et al., 2023). From a microscopic traffic flow dynamics perspective, such abrupt speed drops readily generate upstream-propagating longitudinal traffic shockwaves, thereby exponentially increasing the secondary rear-end collision risk for following vehicles (Zheng et al., 2013). In contrast, although continuous visual deceleration markings lack instantaneous mandatory awakening capabilities, they are more likely to guide drivers into forming a smooth, gradual deceleration maneuver. Therefore, the trade-off boundary between “macroscopic speed compliance” and “microscopic control smoothness” across different warning modalities, as well as the deep interaction effects of visual–auditory multimodal superimposition, still lack systematic deconstruction based on high-frequency continuous trajectory data.

To bridge this research gap, this study leverages a high-fidelity driving simulator platform to construct a baseline Standard Configuration Group strictly adhering to current traffic engineering codes, alongside an Enhanced Configuration Group with continuous markings and an Augmented Warning Group incorporating multimodal visual–auditory alerts. The objective is to systematically investigate the spatiotemporal intervention effects of different warning modalities on drivers’ continuous speed-control behaviors. By exploiting high-frequency vehicle trajectory data and second-order dynamic indicators, this study aims to reveal whether and how different warning configurations influence not only final speed-control outcomes but also the microscopic deceleration process, how these effects vary with spatial proximity to the work zone, and whether the observed intervention effects remain after accounting for individual driving heterogeneity. The findings provide quantitative evidence and theoretical support for the spatially differentiated design of future multimodal active warning systems in highway work zones.

## 2 Methods

### 2.1 Participants

Determining an appropriate sample size is important for ensuring the reliability of driving simulator experiments. Previous microscopic driving-behavior studies focusing on traffic-control and warning facilities have typically employed 20–43 valid participants (Huang et al., 2020; Li et al., 2013). Accordingly, the present study recruited 41 valid participants, which is within the upper range of sample sizes reported in related driving simulator studies. To further assess the adequacy of the sample size, a power analysis was conducted using G*Power 3.1 for a repeated-measures within-subject design with three warning scenarios. Assuming a medium effect size (*f* = 0.25), a significance level of α = 0.05, statistical power of 0.80, a correlation of 0.50 among repeated measures, and a nonsphericity correction of 1.0, the minimum required sample size was 28 participants. Therefore, the final sample of 41 valid participants exceeded this requirement and was considered sufficient for the planned within-subject comparisons.

To mitigate potential confounding effects related to participants’ physical condition and alertness, a brief self-report health screening was conducted before the experiment. Participants were required to have normal or corrected-to-normal vision and to report no physical or neurological conditions that could impair driving performance. Their current level of sleepiness was additionally assessed using the 9-point Karolinska Sleepiness Scale (KSS), ranging from 1 (extremely alert) to 9 (very sleepy, requiring great effort to keep awake) (Åkerstedt & Gillberg, 1990). Participants also confirmed that they were physically and mentally fit to complete the driving task and reported any current discomfort or condition that might affect driving performance.

### 2.2 Experimental apparatus

This study utilized the Innosimulation CDS compact driving simulator as the core experimental platform (Figure 1). The left-hand drive, automatic transmission cabin is equipped with realistic operational components, while both the steering wheel and pedals are integrated with a force-feedback system to ensure high-fidelity physical interactions. The visual feedback system consists of three 55-inch high-definition LED screens, providing a resolution of 3,840 × 2,160 with a 100° horizontal and 25° vertical field of view (FOV). The underlying simulation environment is driven by a networked cluster of four high-performance computers (equipped with RTX 3080 GPUs and Intel Core i7 processors), guaranteeing zero-latency synchronization between precise vehicle dynamics data acquisition and multimodal audio-visual rendering.

**Figure 1:**
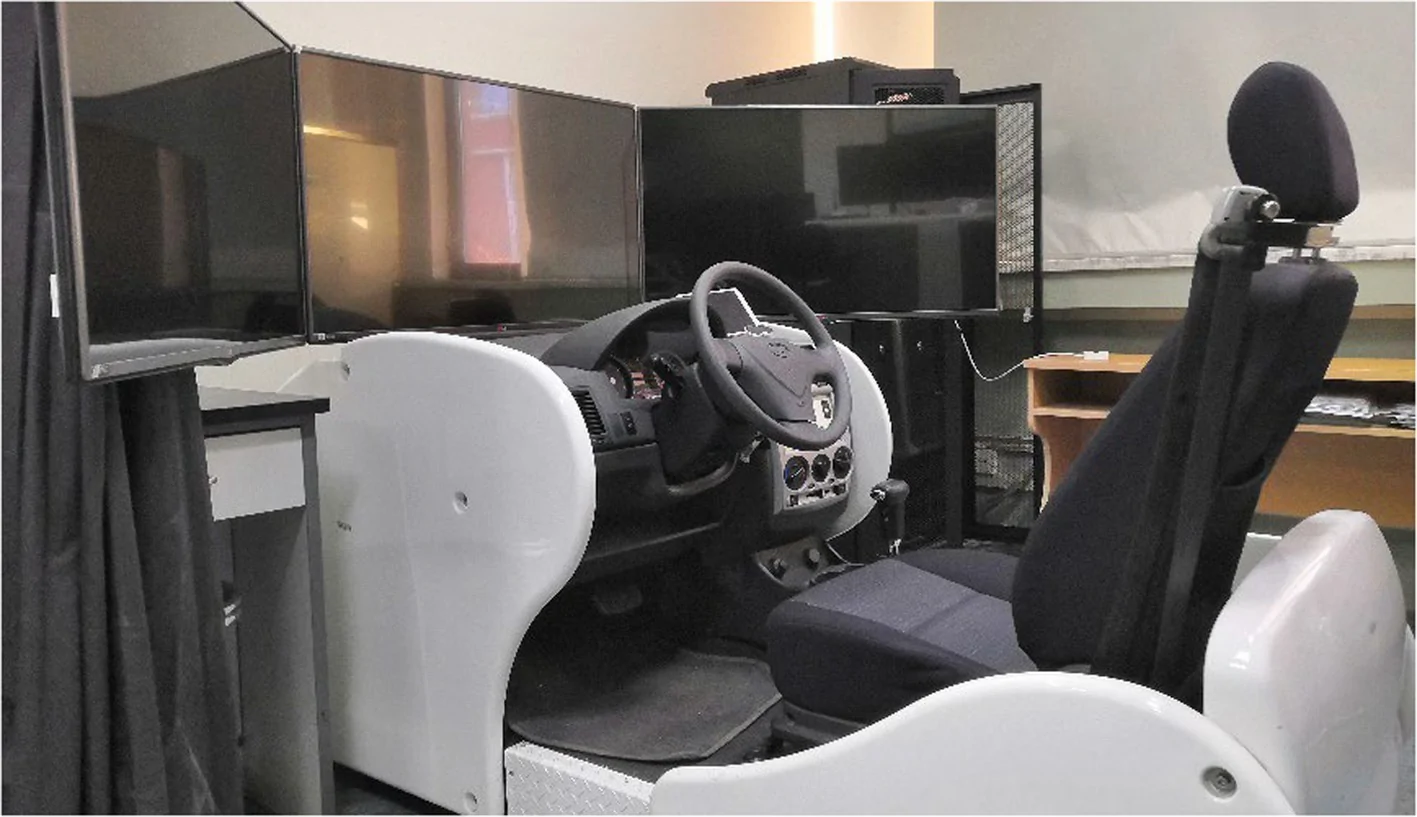
Experimental equipment.

### 2.3 Scenario design

To systematically investigate the effects of different warning modalities on drivers’ deceleration behaviors when approaching work zones, this study selected the reconstruction and expansion section of the G30 Lianyungang–Khorgas Expressway (Xinjiang, China) as the real-world prototype. The G30 Expressway is a major east–west national trunk route and one of the principal highway corridors connecting Xinjiang with other regions of China. Its reconstruction and expansion involve traffic-maintained work zones with successive warning and speed-control requirements, making it a representative setting for examining drivers’ continuous deceleration responses when approaching highway work zones. A highly realistic test track was therefore constructed using the driving simulator, featuring three progressive warning intervention scenarios (Figure 2).

(1) Scenario A (Standard Configuration Group) (Figure 3): Strictly adhering to current traffic engineering specifications, this baseline scenario only deploys static “Road Work Ahead” warning signs and stepped speed limit signs (80 km/h and 60 km/h) at the beginning of the advance warning and transition areas. No special physical interventions are applied to the road surface.
(2) Scenario B (Enhanced Configuration Group) (Figure 4): Building upon Scenario A, this group incorporates continuous transverse colored markings and longitudinal deceleration markings on the road surface. This design aims to induce a more proactive and gradual deceleration maneuver through the optical “space compression effect.”
(3) Scenario C (Augmented Warning Group) (Figure 5): Superimposing an auditory warning dimension onto the visual interventions of Scenario B, roadside directional loudspeakers are deployed to prompt drivers to decelerate. The triggering mechanism for the voice alerts is strictly based on the vehicle’s absolute spatial coordinates. The sound pressure level is meticulously maintained between 75 and 80 dB to ensure effective instantaneous awakening without causing physiological discomfort or excessive startle responses.

**Figure 2:**
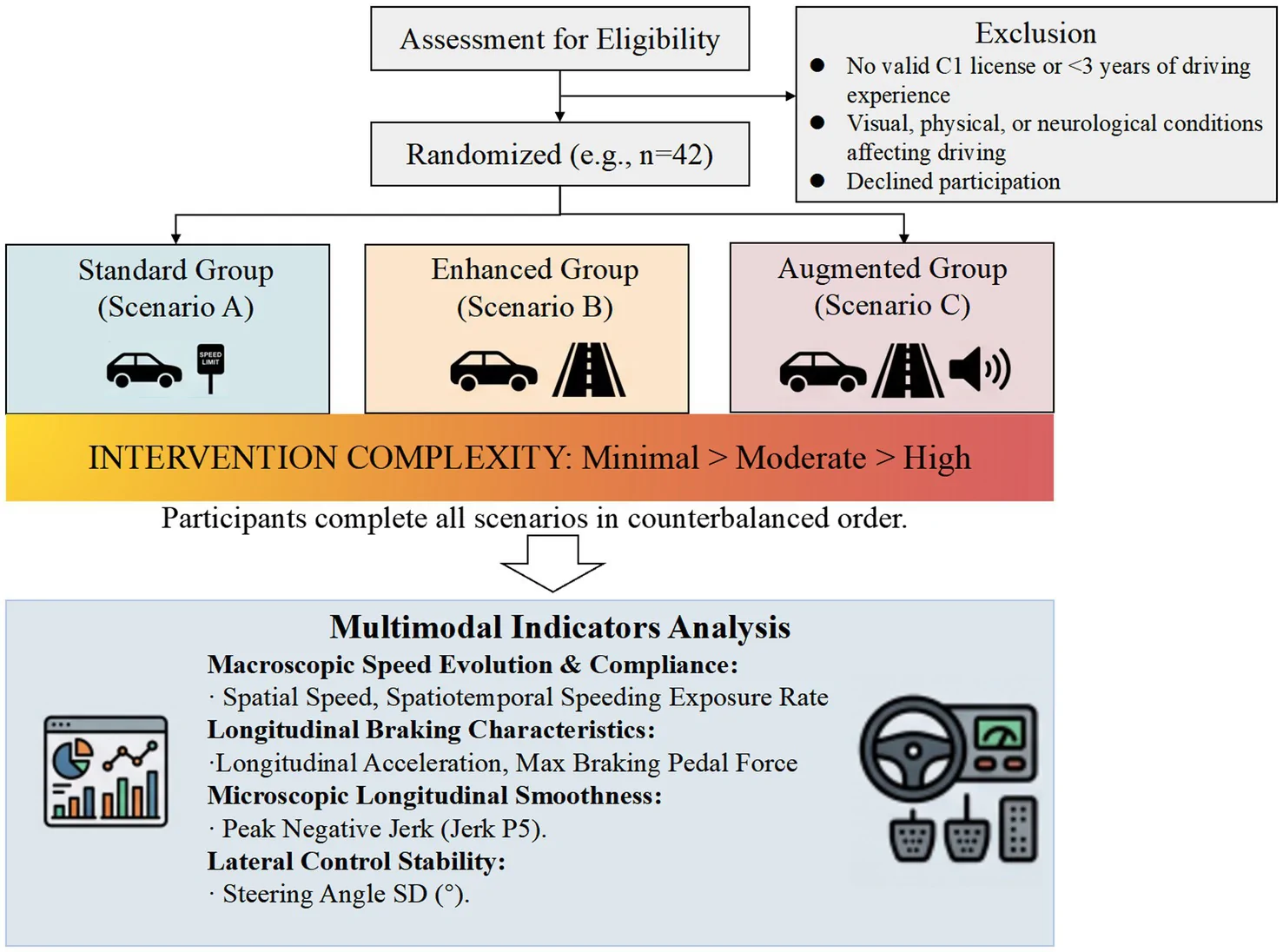
Experimental design.

**Figure 3:**
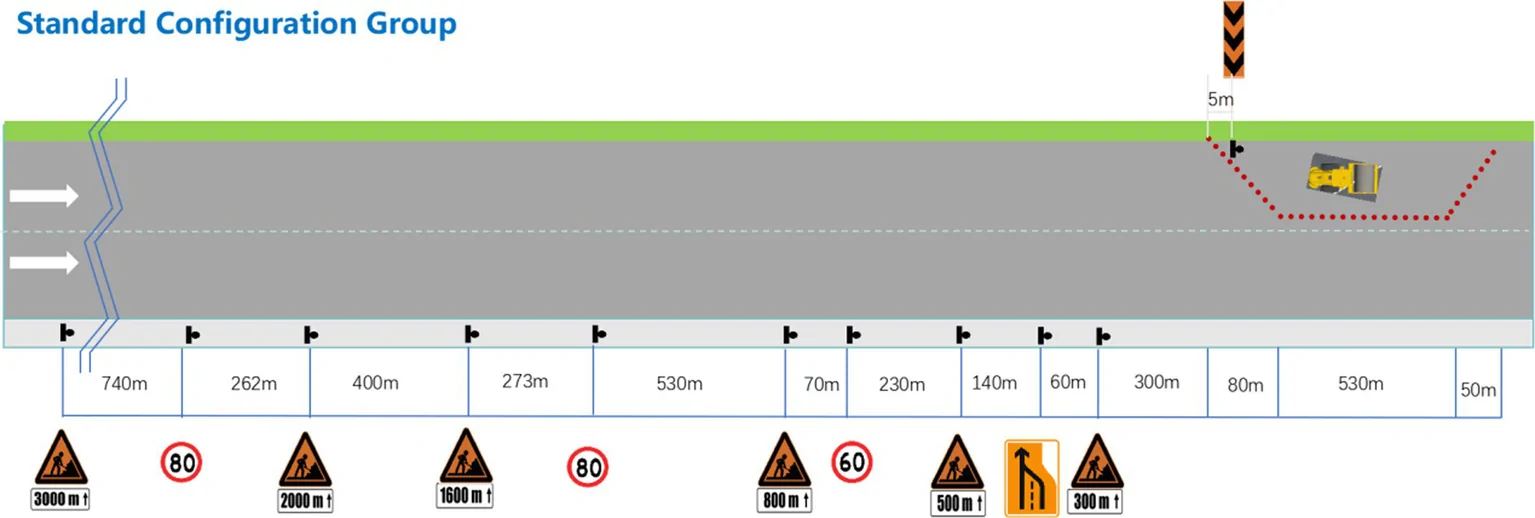
Schematic layout of scenario A.

**Figure 4:**
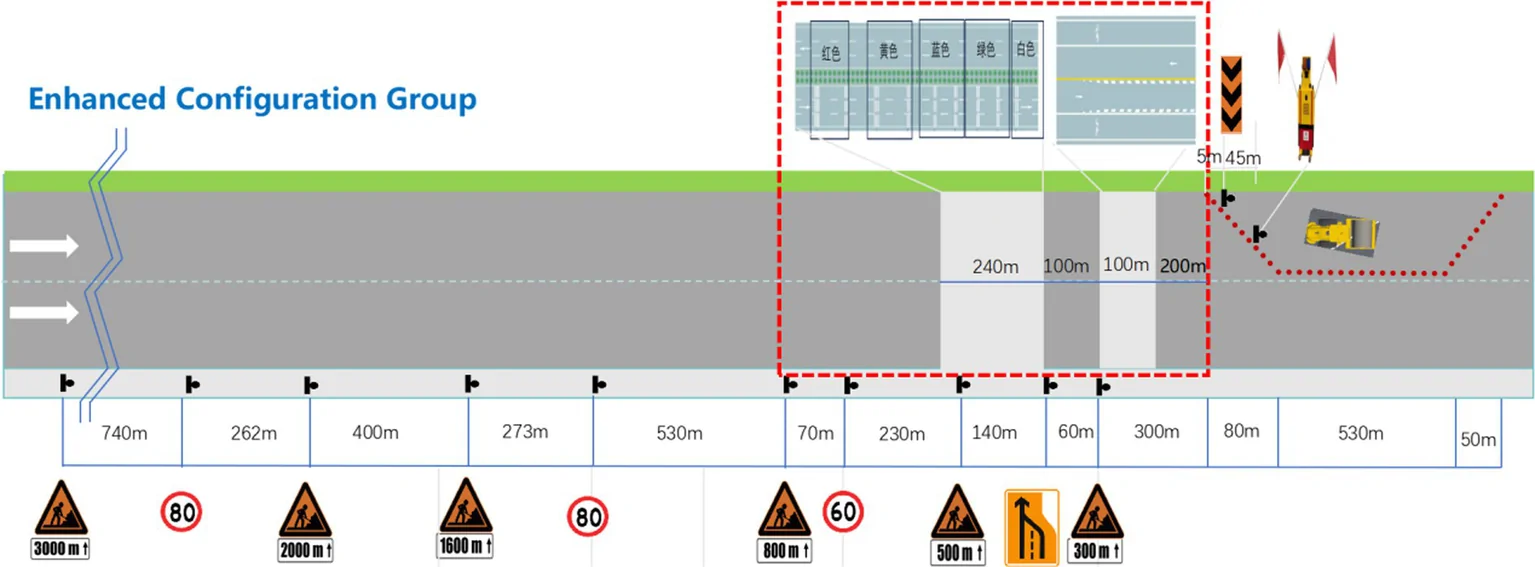
Schematic layout of scenario B.

**Figure 5:**
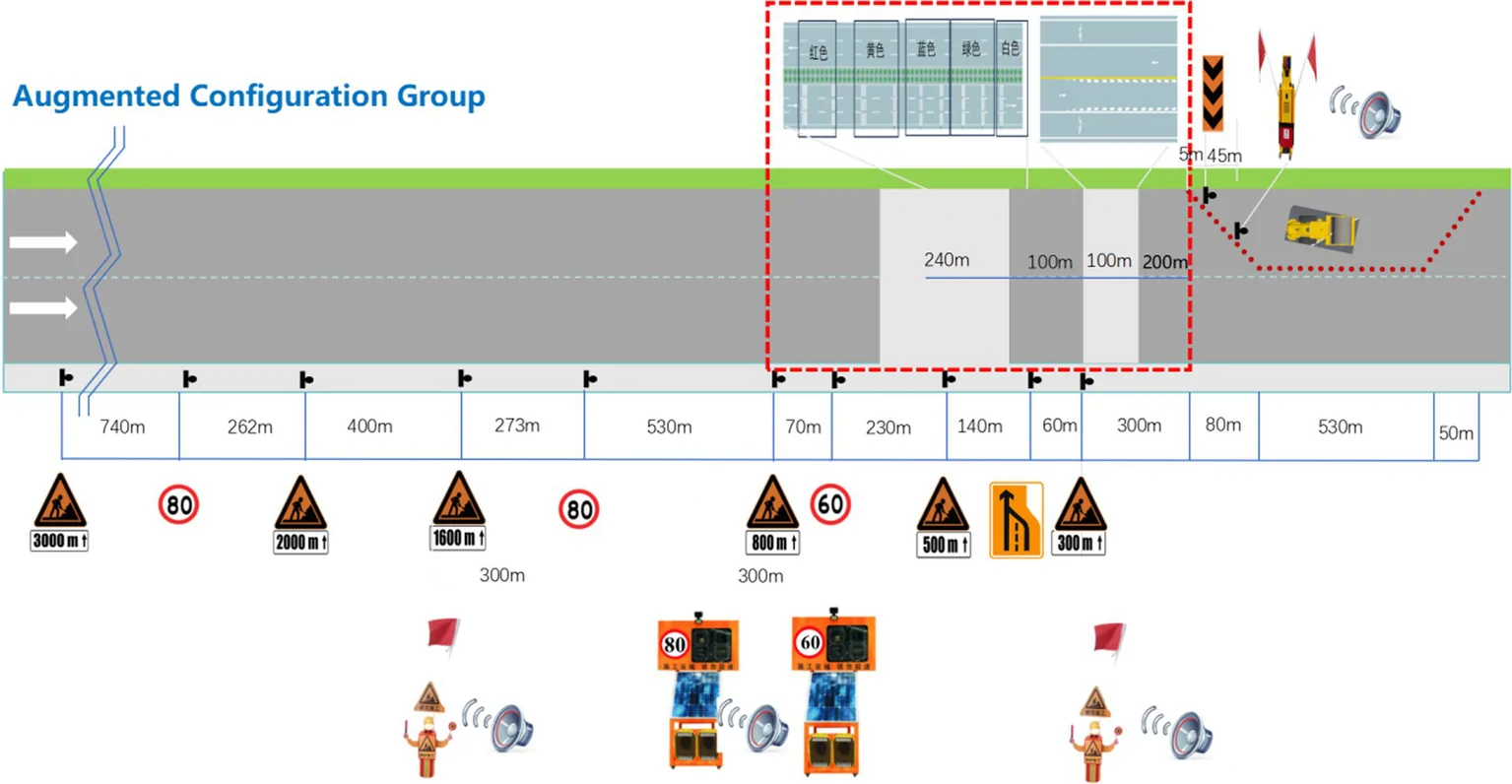
Schematic layout of scenario C.

Four discrete voice alerts (Voice 1–Voice 4) were configured in Scenario C, with each alert triggered once when the vehicle reached its predefined spatial location. Voice 1 and Voice 2 were triggered at approximately 2,390 m and 2,990 m, respectively, while Voice 3 was triggered at approximately 3,290 m near the transition to the 60 km/h core control zone. Voice 4 was positioned further downstream within the final approach to the work-zone boundary, as illustrated in Figure 5. These warning points were progressively distributed along the work-zone approach to correspond to successive traffic-control stages. The upstream alerts were intended to provide advance attention reminders during relatively low-workload cruising, whereas the downstream alerts reinforced the impending speed-control and work-zone constraints. Multiple discrete alerts were therefore used to provide staged rather than continuous auditory guidance and to examine whether the effectiveness of auditory warnings varied with proximity to the work zone.

### 2.4 Experimental procedure

To ensure data validity and eliminate learning interference caused by order effects, a strict standardized experimental protocol was implemented, consisting of the following steps:

(1) Familiarization and Pre-experiment: Prior to the formal trials, participants engaged in a 5-min adaptive driving session in a conventional highway scenario unrelated to the test track. This stage aimed to help participants establish muscle memory for the simulator’s steering and pedal force-feedback. During this period, participants completed the demographic questionnaire and the pre-experiment health screening, and rated their current level of sleepiness using the KSS.
(2) Standardized Instruction Briefing: The experimenter read a standardized script to participants, detailing the driving tasks and safety precautions. Participants were explicitly instructed to “drive completely in accordance with their daily naturalistic driving habits and subjective risk judgments,” strictly adhere to the guidance of traffic signs along the route (including speed limit and lane change information), and report any physical discomfort, such as simulator sickness, to terminate the experiment immediately if necessary.
(3) Formal Experiment and Scenario Randomization: Participants then completed the driving tasks across the three warning scenarios (Scenarios A, B, and C). To effectively eradicate potential learning effects and the accumulation of driving fatigue, the presentation order of the three scenarios was structured using a rigorous randomized counterbalancing design.
(4) Data Archiving and Post-experiment Assessment: Upon completion of all driving tasks, the system automatically archived the high-frequency trajectory and operational behavioral data. Participants exited the cabin and completed the KSS again to assess their post-experiment level of sleepiness. They then completed a brief structured post-driving questionnaire to evaluate their perceived immersion and the realism of the simulator environment and vehicle-control feedback.

### 2.5 Experimental indicators

(1) Macroscopic speed evolution and compliance.

Spatial speed and deceleration magnitude: Extract the average speed within each spatial phase and the absolute speed difference from the initial free-flow cross-section to the start of the work zone. This is used to evaluate the macroscopic compliance effect of the vehicle’s overall kinetic energy reduction (Tang et al., 2026).

Spatiotemporal speeding exposure rate: Defined as the ratio of speeding trajectory points to the total valid trajectory points within a specific control zone. Compared to discrete cross-sectional speeds, this indicator can accurately quantify the spatiotemporal cumulative effect of speeding behaviors, reflecting drivers’ speed adaptation and vigilance relaxation under long-distance guidance (Ugan et al., 2023).

(2) Longitudinal braking characteristics.

Longitudinal acceleration and maximum deceleration: Extract the continuous sequence of vehicle longitudinal acceleration to depict the deceleration fluctuation profile, and calculate the maximum longitudinal deceleration (negative peak) for each phase (Lee et al., 2002). This peak represents the physical braking limit and rigid constraint boundary when the vehicle attempts to avoid collisions.

Maximum braking pedal force (P95, %): Extract the 95th percentile peak of the brake pedal depression depth within each phase, which is used to directly map the driver’s subjective ultimate braking intent when confronting warning stimuli or speed limit constraints.

(3) Microscopic Longitudinal Smoothness.

Peak negative jerk (Jerk P5): The rate of change of acceleration over time is adopted as the core second-order dynamic indicator of longitudinal smoothness (Bagdadi, 2013). To filter high-frequency mechanical noise, the 5th percentile of the negative jerk distribution is extracted. A smaller absolute value of this indicator suggests a smoother deceleration process, thereby indicating a lower risk of inducing longitudinal traffic shockwaves and secondary rear-end collisions.

(4) Lateral control stability.

Steering angle standard deviation (Steering Angle SD, °): Extract the standard deviation of the vehicle’s steering wheel angle within each phase (Engström et al., 2005). This indicator is utilized to quantify the lateral control workload fluctuation and trajectory instability risk triggered by rigid physical boundaries as vehicles approach and traverse work zone lane bottlenecks.

### 2.6 Spatial phase definition

For the subsequent phase-based analyses, the work-zone approach corridor was divided into four spatial phases: Phase I (1,015–1,755 m), Phase II (1,755–2,690 m), Phase III (2,690–3,290 m), and Phase IV (3,290–4,015 m). The phase boundaries were determined primarily according to roadway control transitions and physical traffic-control facilities common to all three scenarios, rather than by individual auditory-warning trigger points. This ensured that identical spatial segments were used for cross-scenario comparisons. Accordingly, Voice 1 (approximately 2,390 m) was treated as an intervention event within Phase II rather than as an independent phase boundary. Voice 2 was similarly located within Phase III, whereas Voice 3 coincided with the 3,290-m transition to the 60 km/h core control zone, and Voice 4 was located within Phase IV.

### 2.7 Linear mixed-effects modeling considering individual heterogeneity

Given the within-subject design of this experiment, each participant traversed all three warning scenarios. Consequently, the microscopic maneuvering behaviors of the same driver across different intervention conditions exhibit intrinsic correlations. Traditional Analysis of Variance (ANOVA), which relies strictly on the independent and identically distributed (i.i.d.) assumption, tends to obscure baseline variations caused by inherent driving styles, thereby confounding the true experimental effects. To precisely isolate unobserved individual heterogeneity, this study developed a Linear Mixed-Effects Model (LMM) incorporating spatiotemporal interaction terms to deeply deconstruct the microscopic dynamic and compliance indicators.

By introducing a participant-level random intercept, the LMM effectively controls for baseline operational variances among individual drivers, thereby enabling an unadulterated estimation of the empirical intervention efficacy exerted by different warning modalities (fixed effects). The mathematical formulation of the model is expressed as follows:

$$Y_{\mathrm{ij}}=\beta_{0}+\beta_{1}S_{B,\mathrm{ij}}+\beta_{2}S_{C,\mathrm{ij}}+\sum \gamma P_{\mathrm{ij}}+\sum \sum \delta_{\mathrm{kp}}(S_{k,\mathrm{ij}}\times P_{p,\mathrm{ij}})+\mu_{i}+\varepsilon_{\mathrm{ij}}$$

$Y_{\mathrm{ij}}$ denotes the microscopic dynamic or compliance indicator of participant i in the j-th observation; $\beta_{0}$ represents the global fixed intercept for the baseline group (Scenario A); $S_{B,\mathrm{ij}}$ and $S_{C,\mathrm{ij}}$ are dummy variables representing the enhanced visual marking group (Scenario B) and the augmented visual–auditory warning group (Scenario C), with $\beta_{1}$ and $\beta_{2}$ as their corresponding fixed main effect coefficients, respectively; $\text{Phas}e_{\mathrm{ij}}$ represents the vector of spatial phase variables, and γ denotes the vector of fixed effect coefficients controlling for different spatial zones; $S_{k,\mathrm{ij}}\times P_{p,\mathrm{ij}}$ denotes the spatiotemporal interaction terms, where $\delta_{\mathrm{kp}}$ precisely captures the evolutionary intervention efficacy of a specific warning modality within a specific spatial zone; $\mu_{i}$ signifies the random intercept for driver i, capturing the extent to which the participant’s inherent driving aggressiveness or baseline performance deviates from the global mean; $\varepsilon_{\mathrm{ij}}$ is the normally distributed residual term. Furthermore, to statistically validate the absolute necessity of incorporating random effects, the Intraclass Correlation Coefficient (ICC) was calculated to quantify the proportion of total variance in microscopic operations attributable to inherent inter-individual differences.

Potential multicollinearity among the fixed effects was assessed using Variance Inflation Factors (VIFs) calculated from the fixed-effect design matrix, including the warning-scenario indicators, spatial-phase indicators, and their interaction terms. Because VIF cutoffs are empirical rules of thumb rather than universally fixed criteria, the diagnostics were interpreted with reference to both the conservative threshold of 5 and the commonly used higher threshold of 10 (Akinwande et al., 2015; O’brien, 2007).

## 3 Results

### 3.1 Continuous spatial evolution analysis of microscopic driving behavior

(1) Speed distribution.

As shown in Figure 6, the speed distribution curves provide a descriptive comparison of the operating-speed patterns under the three warning scenarios. The speed distribution of Scenario A (Standard Configuration Group), which relies solely on static signs, is relatively dispersed, reflecting greater variability in driving behavior. Scenario B, which incorporates deceleration markings, exhibits a more concentrated speed distribution, although the overall speed remains relatively high. In contrast, Scenario C, which superimposes directional voice prompts, shows a noticeable leftward shift in the speed distribution and an earlier increase in cumulative probability. These descriptive patterns suggest that the visual–auditory intervention is associated with lower overall operating speeds and a stronger apparent deceleration tendency than the other two scenarios. Statistical significance is not inferred from these distribution curves; inferential comparisons are reported in the subsequent spatial analyses.

(2) Spatiotemporal evolution of macroscopic speed.

**Figure 6:**
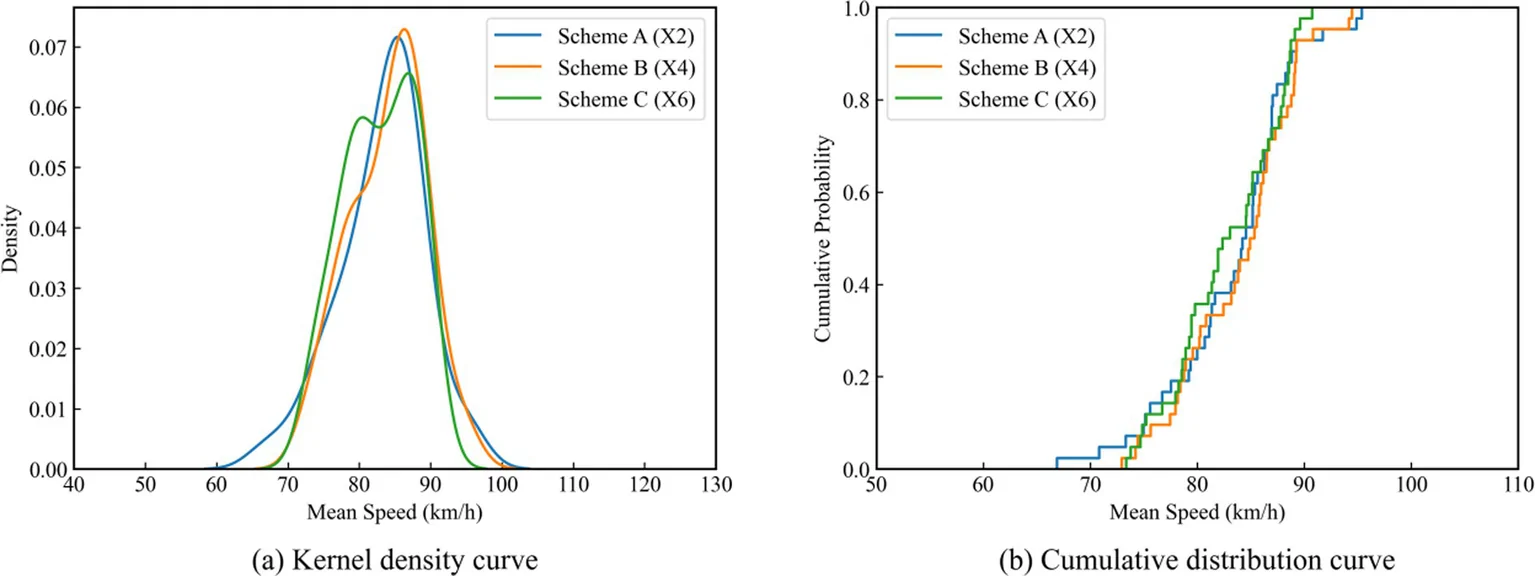
Speed distribution curves: **(a)** kernel density curve; **(b)** cumulative distribution curve.

Figure 7 visualizes the spatial evolution trajectories of vehicle operating speeds under continuous multimodal warning interventions. Following the initial braking adaptation, vehicles entered the mid-segment cruising zone. Although the Augmented Warning Group (Scenario C) triggered isolated auditory alerts at approximately 2,390 m (Voice 1) and 2,990 m (Voice 2), the speed evolution curves of the three groups highly overlapped (*p* > 0.05). This indicates that in far-field low-workload sections, isolated auditory interventions are easily adapted to by drivers and fail to induce statistically significant deceleration divergence. As vehicles approached the 60 km/h speed limit sign and entered the intensive intervention zone (“Voice 3 + Markings 1 + Voice 4”), constrained by the mandatory speed limit, all three groups synchronously executed secondary decelerations, smoothly transitioning to approximately 70 km/h. The inter-group differences within this transition phase remained non-significant (*p* > 0.05). However, in the high-visual-workload bottleneck zone immediately preceding the work zone start point (i.e., the second continuous markings section, Markings 2, from 3,700 m to 3,900 m), a highly significant inter-group divergence in speed evolution emerged (*p* < 0.05). Within this critical interval, the deceleration trend of the Standard Configuration Group (Scenario A) stagnated and even exhibited a slight rebound (stabilizing at around 55 km/h), exposing obvious driver laxity. In stark contrast, the speeds of the Enhanced Configuration Group (Scenario B) and the Augmented Warning Group (Scenario C) were significantly and continuously suppressed. Scenario C, in particular, saw its average speed plunge to an overall minimum of 50 km/h, demonstrating a highly significant localized speed control superiority prior to ultimately entering the core work zone boundary at 4015 m.

**Figure 7:**
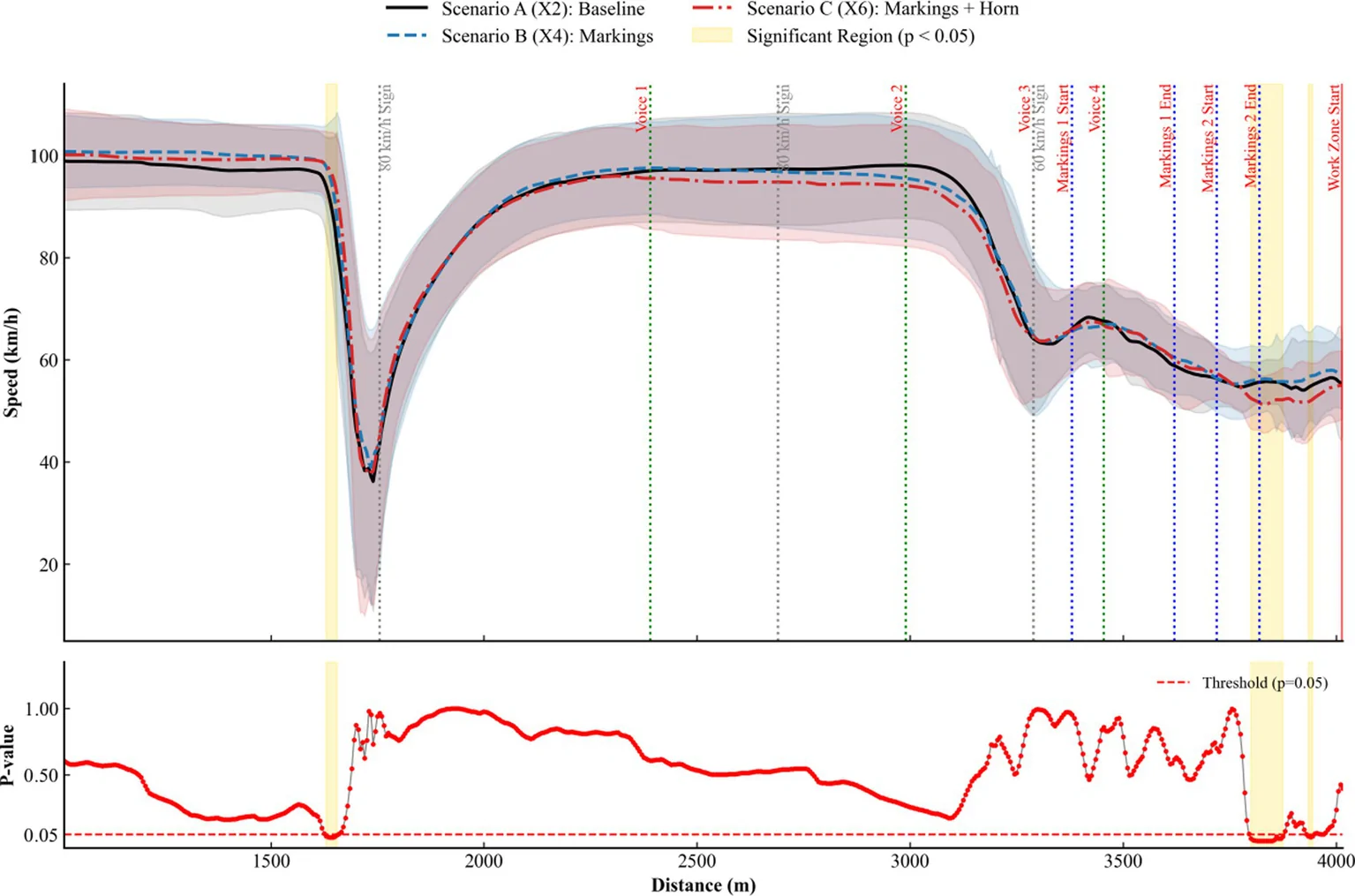
Mean speed trajectories across the three experimental scenarios for all participants.

(3) Second-order Dynamic Evolution of Microscopic Smoothness (Jerk).

Figure 8 profoundly reveals the spatiotemporal intervention characteristics of different warning modalities on the vehicle’s microscopic longitudinal control smoothness. In the extended mid-segment cruising zone (isolated trigger points for Voice 1 and Voice 2), the longitudinal jerk curves of all three groups remained stable near 0 m/s^3^, showing no significant inter-group differences (*p* > 0.05). This indicates that in far-field, low-workload sections, isolated auditory stimuli functioned solely as mild informational cues without inducing abrupt mechanical braking, thereby preserving baseline driving smoothness. As vehicles entered the intensive warning zone (“Voice 3 + Markings 1 + Voice 4”), the fluctuation frequency of jerk in all groups increased significantly (*p* < 0.05). Notably, however, the peak negative jerk values within this region were effectively constrained within the human comfort dynamic threshold of −1.5 m/s^3^. This confirms that the multimodal visual–auditory coordinated intervention successfully reshaped the deceleration profile during this critical phase. By progressively awakening drivers’ attention, it effectively averted panic hard braking induced by sudden speed limit constraints. Finally, in the second continuous markings section (Markings 2) immediately preceding the work zone start point, microscopic jerk exhibited highly significant localized evolutionary divergence (*p* < 0.05). In stark contrast to the single, deep hard braking triggered by a lack of psychological preparedness during initial encounters with speed limits, the continuous markings in this high-risk zone successfully transformed the warning groups’ coarse deceleration behaviors into “high-frequency, low-amplitude” micro-pedal corrections. This further substantiates that within the ultimate critical distance before entering the geometric bottleneck, continuous visual intervention can substantially expand the safety margin of microscopic dynamics through smooth, high-density visual guidance, without triggering high-risk longitudinal traffic shockwaves.

(4) Continuous spatial evolution of longitudinal acceleration.

**Figure 8:**
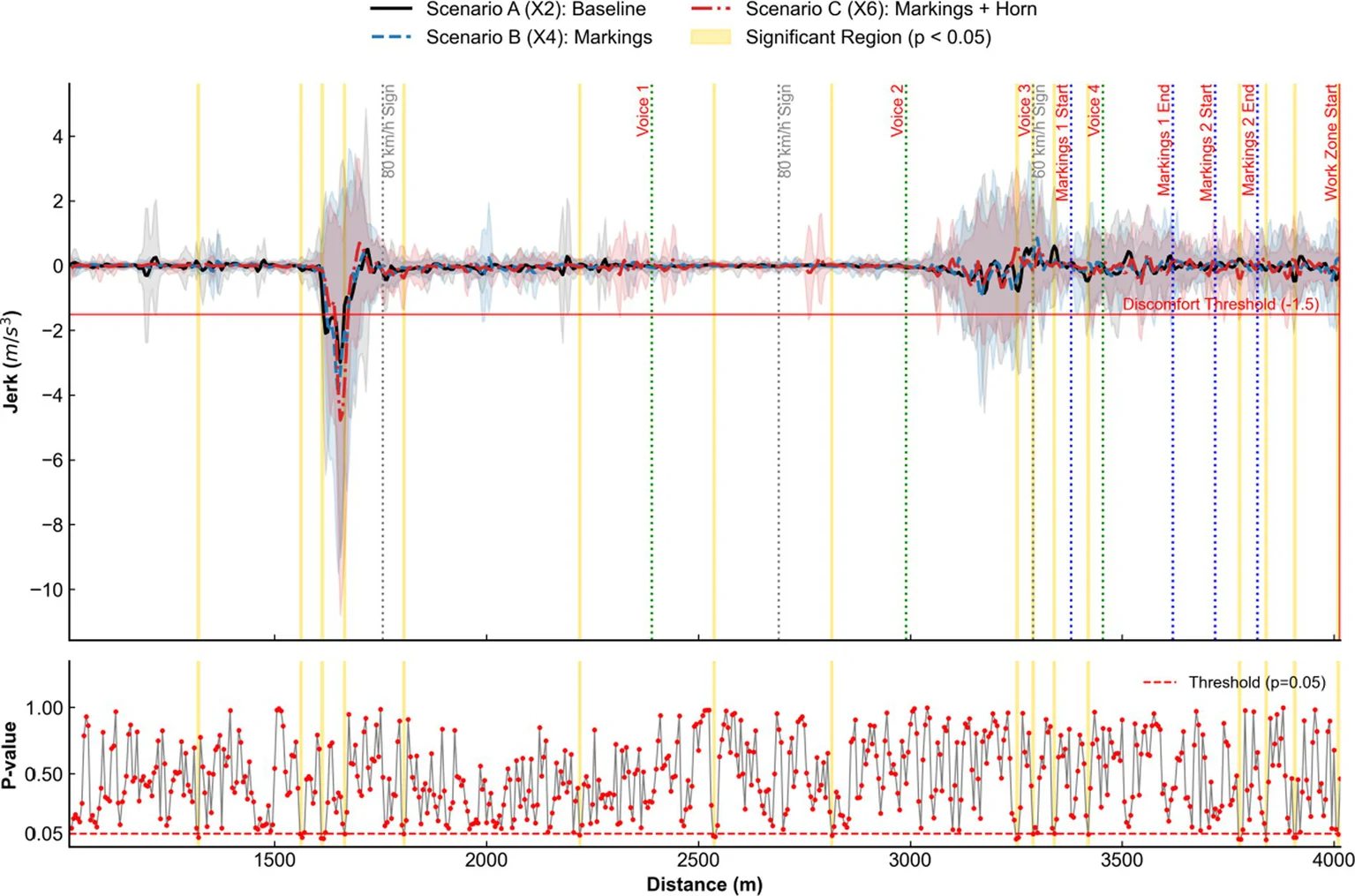
Jerk trends and significance test results over approach distance across different experimental scenarios.

Figure 9 visually unfolds the specific regulatory characteristics of multimodal warning facilities on drivers’ continuous longitudinal acceleration and deceleration behaviors. In the extended mid-segment cruising zone (isolated trigger points for Voice 1 and Voice 2), the longitudinal acceleration curves of all three groups oscillated stably around the 0 m/s^2^ baseline, triggering no statistically significant inter-group differences. This further corroborates that in far-field open sections with low workload, isolated auditory stimuli are insufficient to elicit substantial proactive physical braking behaviors from drivers. As vehicles navigated into the intensive composite intervention zone (“Voice 3 + Markings 1 + Voice 4”), constrained by the impending 60 km/h mandatory speed limit sign, all three groups exhibited a secondary deceleration trough of approximately −1.5 m/s^2^, with no significant differences observed among the groups (*p* > 0.05). This indicates that the multimodal visual–auditory interventions in this region smoothly facilitated the vehicles’ deceleration transition, achieving the speed-control objective without inducing shockwave-triggering excessive braking or hard stops in any specific scenario. However, in the second continuous markings section (Markings 2) immediately approaching the work zone start point, localized microscopic maneuver divergence became highly significant (*p* < 0.05). The acceleration of the baseline group (Scenario A) prematurely recovered to 0 and even exhibited slight positive values, exposing a rear-end-prone “deceleration laxity and micro-acceleration tendency.” Conversely, the warning groups equipped with continuous markings (Scenarios B and C) robustly maintained negative acceleration. This phenomenon profoundly validates that high-density visual interventions at the terminal end can effectively counteract drivers’ vigilance decay and operational inertia following the initial deceleration phase, exerting a sustained and substantial longitudinal braking constraint within the ultimate critical distance before entering the physical work zone.

(5) Microscopic evolution of braking pedal depth.

**Figure 9:**
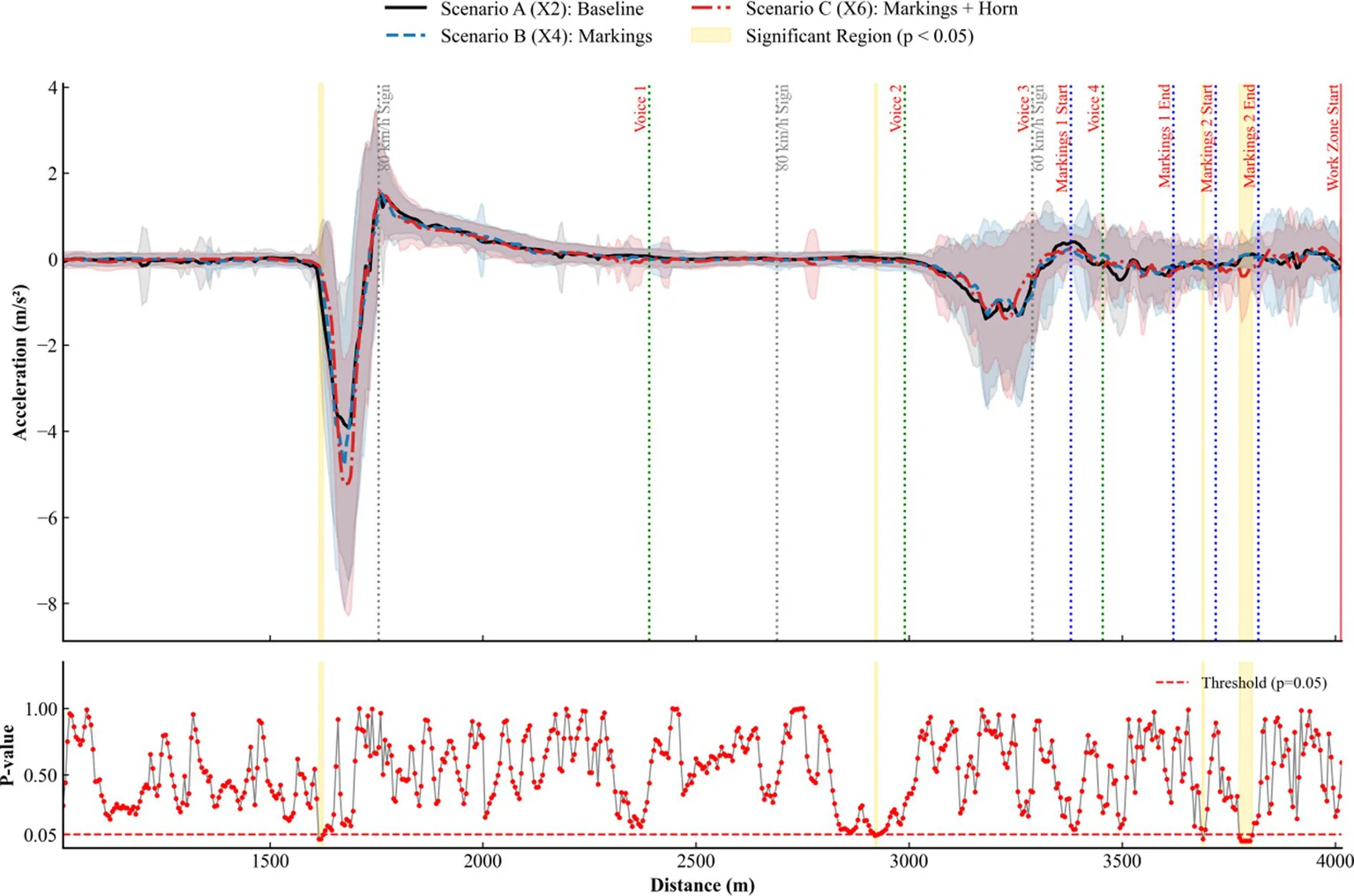
Acceleration trends and significance test results over approach distance across different experimental scenarios.

Figure 10 meticulously delineates the spatial intervention characteristics of different warning modalities on drivers’ proactive braking maneuvers (brake pedal depression depth). In the extended mid-segment cruising zone (isolated trigger points for Voice 1 and Voice 2), the brake pedal depths across all three groups remained stable near 0%, exhibiting no statistically significant differences (*p* > 0.05). This indicates that in far-field open sections lacking continuous physical visual references, isolated auditory stimuli are insufficient to evoke drivers’ substantial active braking intent, making them highly susceptible to cognitive adaptation and information neglect regarding solitary alarms. As vehicles approached the 60 km/h mandatory speed limit sign and entered the intensive intervention zone (“Voice 3 + Markings 1 + Voice 4”), drivers began executing a smooth secondary braking maneuver with an amplitude of approximately 2 to 3%. Within this deceleration transition interval, the multimodal visual–auditory coordinated intervention successfully prompted drivers to adopt a more proactive defensive braking strategy in advance (*p* < 0.05). Crucially, the most fundamental divergence in microscopic maneuvering occurred at the “last line of defense” immediately adjacent to the work zone’s geometric start point—namely, the terminal end of the second continuous markings section (Markings 2) and its extension area (*p* < 0.05). Although the absolute depression depth of the brake pedal at this stage was extremely faint (hovering merely within a 0 to 1% micro-adjustment range), this highly significant localized difference profoundly reveals the sustained constraining efficacy of continuous visual interventions during the ultimate approach phase. Markings 2 successfully counteracted drivers’ operational inertia and vigilance decay following the extended deceleration process, compelling the warning groups (Scenarios B and C) to maintain a highly alert “hovering over the brake” and micro-adjustment state before finally entering the work zone. This mechanism fundamentally constructed a robust safety margin for microscopic maneuvers within the high-risk bottleneck of the final 100 meters.

**Figure 10:**
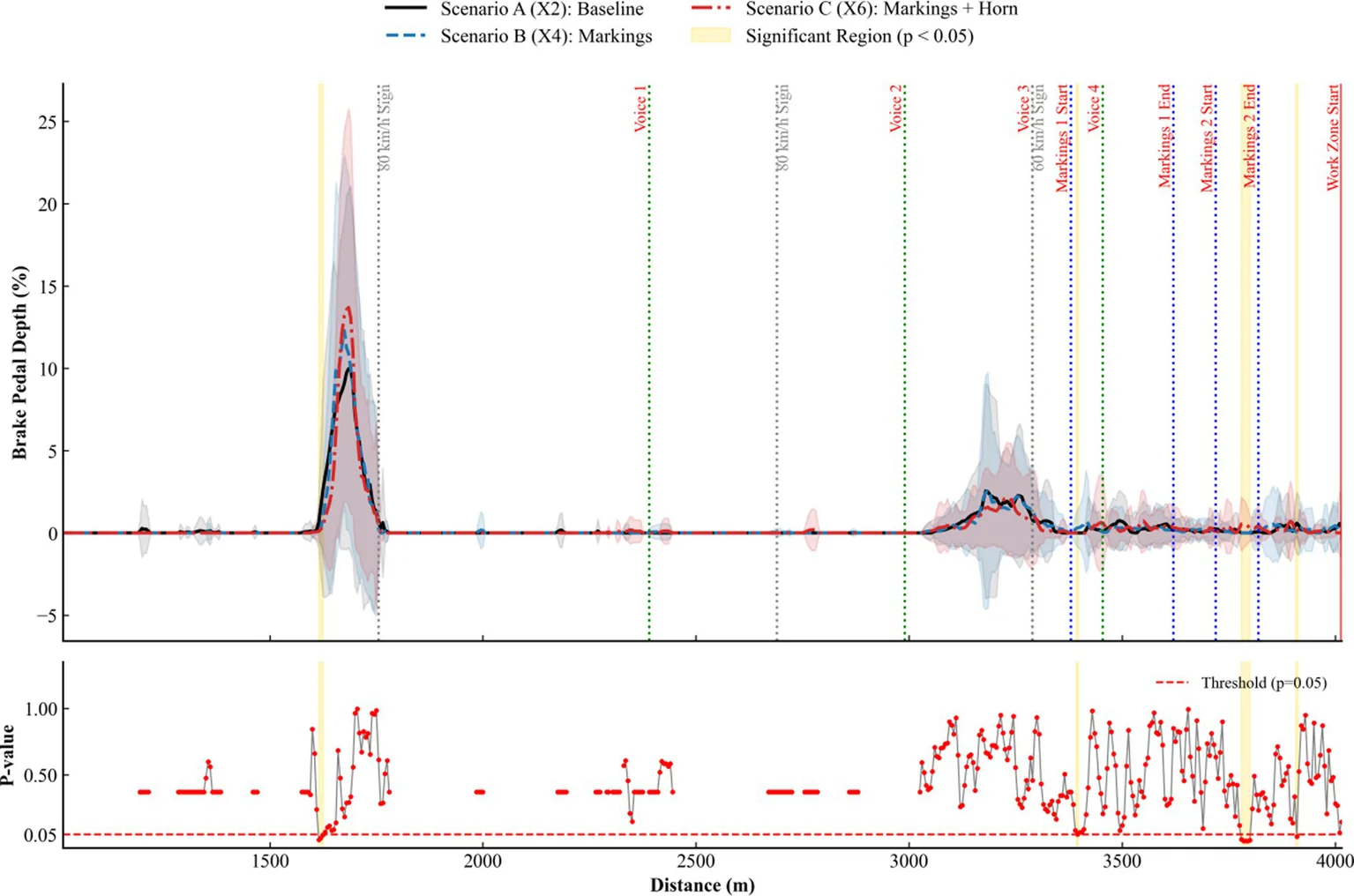
Braking pedal depth trends and significance test results over approach distance across different experimental scenarios.

### 3.2 Macroscopic rigid constraint characteristics of deceleration magnitude and braking intensity

(1) Characteristics of maximum longitudinal deceleration.

Figure 11 extracts the maximum longitudinal deceleration of vehicles across the entire intervention segment. ANOVA results indicate that there is no statistically significant difference in the peak maximum deceleration among the three warning scenarios (*F*(2, 80) = 0.915, *p* = 0.405, η^2^ = 0.022). Notably, the medians of all three groups are highly concentrated between −6.5 m/s^2^ and −7.5 m/s^2^, a value that severely breaches the dynamic comfort threshold of −3.4 m/s^2^ stipulated by AASHTO. This strongly demonstrates that when confronting the rigid compulsion of mandatory speed limits or physical work zone boundaries, drivers will ultimately execute emergency evasive braking of an equivalent depth due to spatial compression. Combining this with the prior dynamic deconstruction of microscopic jerk, it becomes evident that the core safety value of multimodal visual–auditory warnings (Scenarios B and C) does not lie in reducing the absolute physical limit of maximum braking intensity. Rather, by providing early awakening, these warnings prolong the driver’s cognitive reaction window and significantly smooth the transient microscopic control process approaching this braking limit, thereby fundamentally neutralizing high-risk longitudinal traffic shockwaves triggered by a lack of preparedness.

(2) Total deceleration magnitude and kinetic energy dissipation.

**Figure 11:**
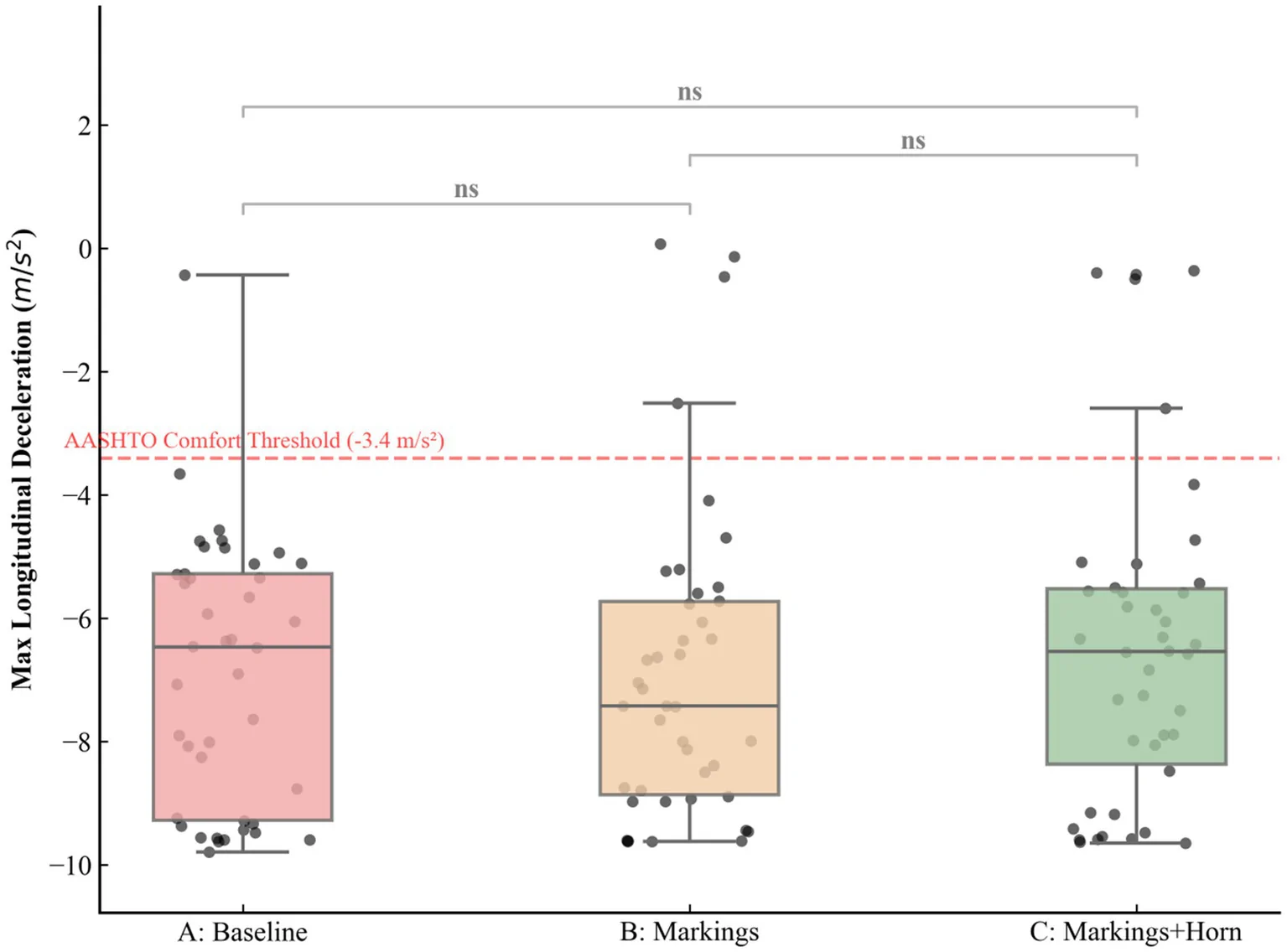
Boxplot and statistical test of maximum longitudinal deceleration under different warning scenarios.

To macroscopically evaluate the impact of warning facilities on the overall kinetic energy dissipation of vehicles, this study extracted the absolute speed difference between the initial free-flow cross-section and the start point of the core work zone. Figure 12 visually presents the spatial distribution characteristics of the total deceleration magnitude under the three intervention scenarios, where the negative values on the vertical axis directly quantify the absolute kinetic energy stripped away by drivers throughout the advance warning and transition zones. One-way ANOVA reveals that the macroscopic cross-sectional deceleration magnitudes across the three scenarios also exhibit no statistically significant differences (*F*(2, 80) = 0.368, *p* = 0.693, η^2^ = 0.009). In terms of absolute data distribution, the deceleration medians of the three vehicle groups highly converge between −42 km/h and −48 km/h, with the core interquartile range (IQR) boxes and scatter patterns highly overlapping. This result profoundly reveals that under the combined constraints of a definitive speed limit target (60 km/h) and physical barricade boundaries, the driver’s overall deceleration task is “rigidly anchored.” Driven by the instinctive baseline of avoiding physical collisions and severe traffic violations, regardless of whether they received high-frequency, multimodal warning stimuli beforehand, drivers must ultimately complete the compliant deceleration at the cost of dissipating an equivalent magnitude of kinetic energy.

**Figure 12:**
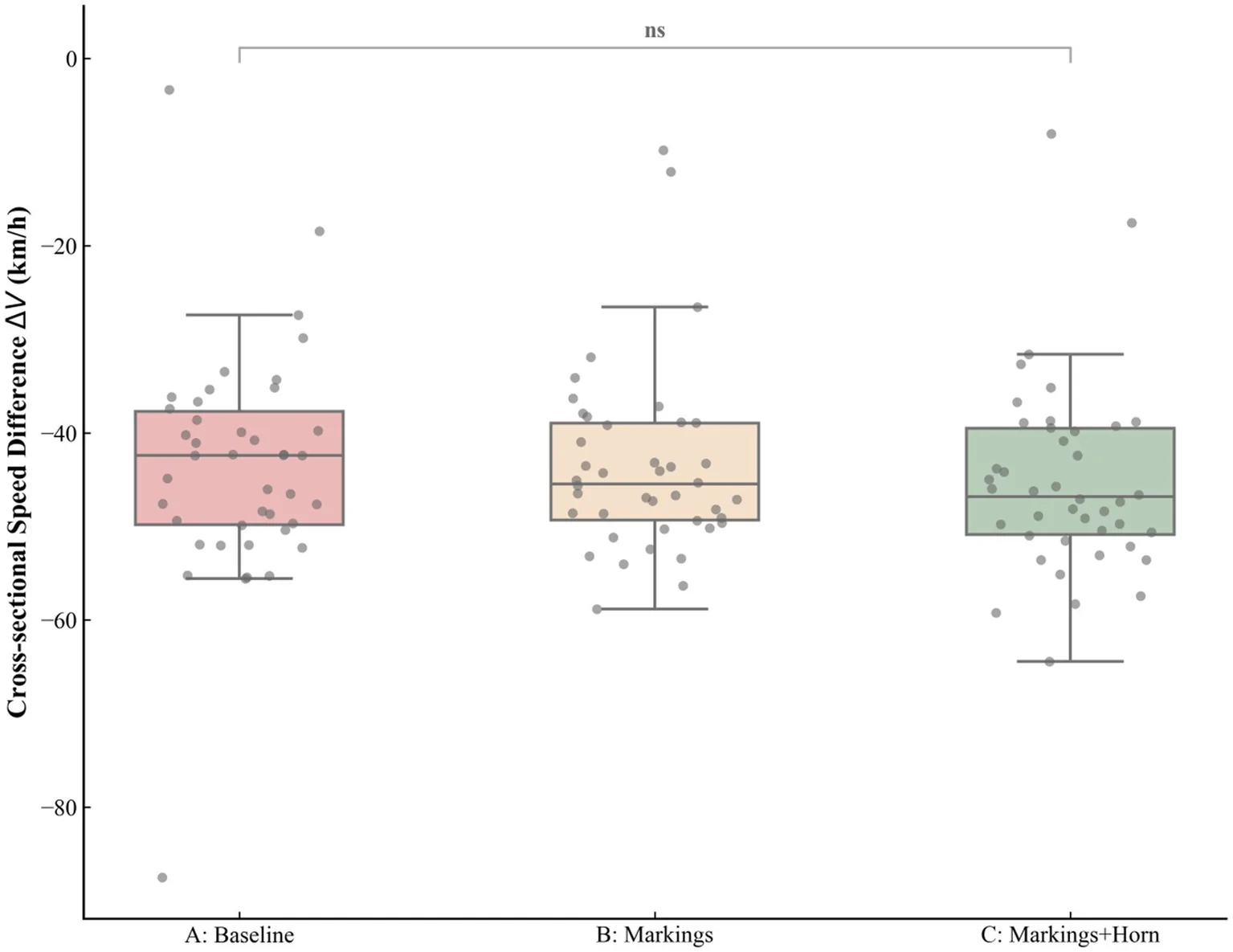
Boxplot and statistical test of total deceleration magnitude under different warning scenarios.

### 3.3 Analysis of longitudinal speed control mechanisms

#### 3.3.1 Spatial configuration of the analysis phases

To facilitate the spatial interpretation of warning effects along the work-zone approach, the analytical corridor was organized into four phases according to roadway control transitions and physical traffic-control facilities common to all experimental scenarios. The detailed spatial configuration and engineering characteristics of each phase are illustrated in Figure 13.

**Figure 13:**
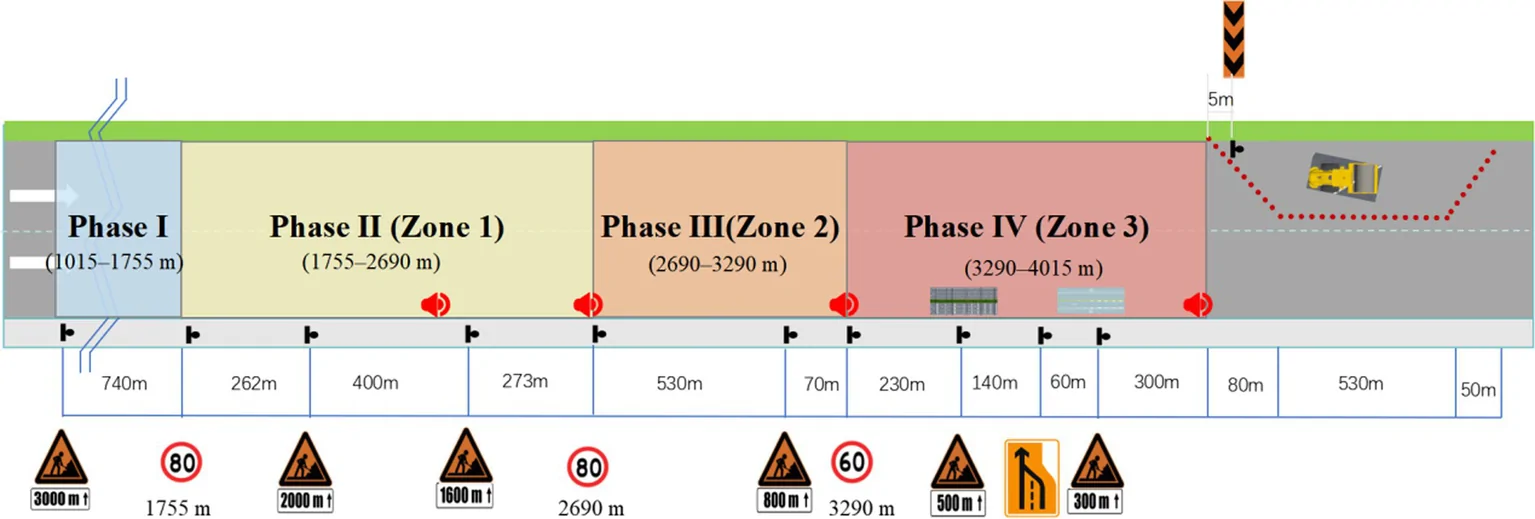
Spatial segmentation of the work zone approach.

##### 3.3.1.1 Phase I: initial speed limit approach zone (1,015 m - 1755 m)

Centered around the first 80 km/h mandatory speed limit sign as the core physical anchor, this phase extends to 1755 m to fully envelope the driver’s initial braking adaptation period following sign recognition. This phase aims to quantify the initial braking intensity and microscopic longitudinal jerk when drivers, cruising under long-distance free-flow conditions, encounter their first physical speed limit constraint.

##### 3.3.1.2 Phase II: Intermediate Cruising & Isolated Voice Zone (1755 m - 2690 m)

This phase covers the extended transition segment between the two 80 km/h speed limit signs. Specifically, within the Augmented Warning Group (Scenario C), this interval includes an isolated voice broadcast (Voice 1, at approx. 2390 m). The engineering rationale for isolating this zone is to examine whether a solitary, far-field auditory stimulus can effectively break drivers’ “automated driving” state and vigilance decay in low-workload open sections lacking continuous visual guidance.

##### 3.3.1.3 Phase III: secondary speed limit transition zone (2,690 m - 3290 m)

Initiating near the second 80 km/h sign and terminating at 3290 m—the trigger point of the 60 km/h core control zone speed limit sign and its accompanying voice alert (Voice 3)—this phase serves as a critical buffer for the traffic flow smoothly transitioning from high- to medium-low-speed regimes. It is primarily used to evaluate drivers’ proactive early deceleration intents prior to recognizing the high-risk work zone ahead.

##### 3.3.1.4 Phase IV: High-Density Multimodal Warning & Core Control Zone (3,290 m - 4015 m)

Immediately preceding the physical start point of the core work zone (4,015 m), this ultimate phase densely embeds the 60 km/h sign, two continuous visual deceleration marking sections (Markings 1 & 2), and an accompanying directional voice prompt (Voice 4). The analytical target of this phase is to profoundly verify whether the cross-modal coordination of “high-density visual space compression” and “auditory semantic awakening” can successfully anchor compliant speeds within the ultimate approach distance, while maximizing maneuvering smoothness and the collision-avoidance safety margin at the microscopic dynamic level.

#### 3.3.2 Bi-directional spatiotemporal deconstruction of multimodal warning intervention efficacy

To systematically examine the spatiotemporal effects of multimodal warning facilities on driving behaviors, repeated-measures ANOVA was conducted from two complementary perspectives: cross-scenario comparisons within each spatial phase and cross-phase comparisons within each warning scenario. The analysis focused on four dimensions: macroscopic speed compliance, braking intensity, longitudinal control smoothness, and lateral trajectory stability (Figure 14). The detailed statistical results are presented in Table 1.

(1) Spatial convergent effect of macroscopic speed compliance.

**Figure 14:**
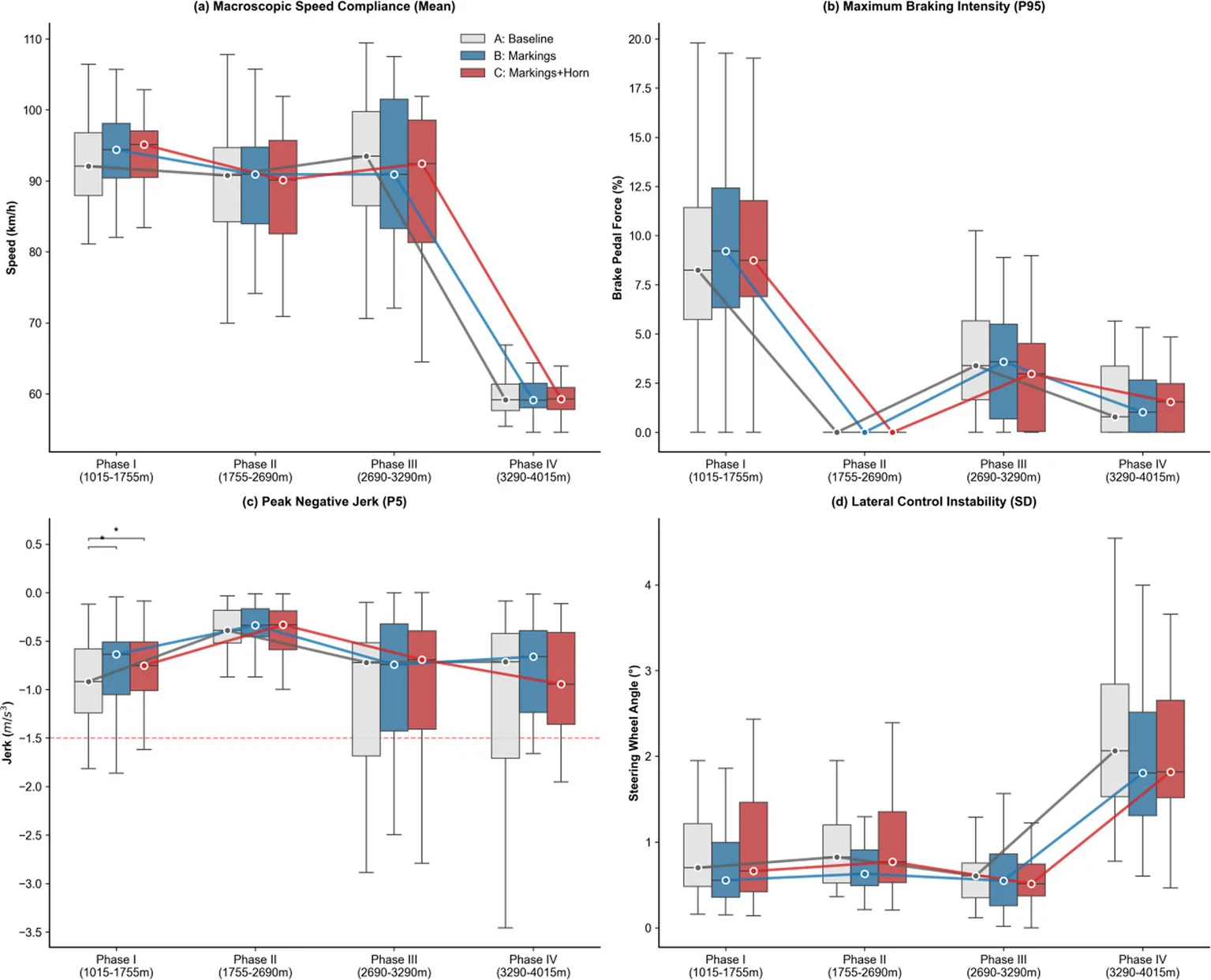
Spatial evolution of driver multi-dimensional behavioral indicators under different work zone warning intervention scenarios: **(a)** macroscopic speed compliance (mean); **(b)** maximum braking intensity (P95); **(c)** peak negative jerk (P5); and **(d)** lateral control instability (SD).

**Table 1:** Spatiotemporal distribution characteristics of vehicle driving states under multimodal warning interventions.

Metric	Scenario	Phase I	Phase II	Phase III	Phase IV	Longitudinal statistics
Mean Speed	A: Baseline	91.11 ± 8.50	89.37 ± 8.78	92.49 ± 9.87	59.85 ± 3.14	*F*(3,120) = 260.637, *p* < 0.001, η^2^ = 0.867
	B: Markings	93.70 ± 6.99	89.92 ± 8.12	91.26 ± 10.56	60.58 ± 3.57	F(3,120) = 236.682, *p* < 0.001, η^2^ = 0.855
	C: Markings+Horn	93.15 ± 7.00	88.38 ± 8.15	88.83 ± 9.94	59.24 ± 3.02	F(3,120) = 235.321, *p* < 0.001, η^2^ = 0.855
Cross-scenario statistics	—	*F*(2,80) = 2.702, *p* = 0.073, η^2^ = 0.063	F(2,80) = 0.935, *p* = 0.397, η^2^ = 0.023	**F(2,80) = 3.381, *p* = 0.039, η**^**2**^ = 0.078*	F(2,80) = 2.928, *p* = 0.059, η^2^ = 0.068	—
Max Braking Force (P95)	A: Baseline	8.92 ± 4.58	0.00 ± 0.00	4.07 ± 4.38	1.70 ± 1.91	*F*(3,120) = 64.536, *p* < 0.001, η^2^ = 0.617
	B: Markings	9.47 ± 5.58	0.04 ± 0.27	3.71 ± 3.51	1.60 ± 1.91	F(3,120) = 57.163, *p* < 0.001, η^2^ = 0.588
	C: Markings+Horn	9.02 ± 4.87	0.01 ± 0.08	2.83 ± 2.55	1.61 ± 1.88	F(3,120) = 74.664, *p* < 0.001, η^2^ = 0.651
Cross-scenario statistics	—	*F*(2,80) = 0.237, *p* = 0.790, η^2^ = 0.006	F(2,80) = 0.717, *p* = 0.491, η^2^ = 0.018	F(2,80) = 1.549, *p* = 0.219, η^2^ = 0.037	F(2,80) = 0.036, *p* = 0.965, η^2^ = 0.001	—
Peak Negative Jerk (P5)	A: Baseline	−1.10 ± 0.88	−0.39 ± 0.23	−1.11 ± 0.89	−1.09 ± 0.93	*F*(3,120) = 12.482, *p* < 0.001, η^2^ = 0.238
	B: Markings	−0.75 ± 0.45	−0.35 ± 0.26	−0.93 ± 0.81	−1.04 ± 0.99	F(3,120) = 9.270, *p* < 0.001, η^2^ = 0.188
	C: Markings+Horn	−0.78 ± 0.43	−0.42 ± 0.37	−0.91 ± 0.71	−0.97 ± 0.72	F(3,120) = 7.046, *p* < 0.001, η^2^ = 0.150
Cross-scenario statistics	—	***F*(2,80) = 4.392, *p* = 0.016, η**^**2**^ = 0.099*	F(2,80) = 1.353, *p* = 0.264, η^2^ = 0.033	F(2,80) = 1.006, *p* = 0.370, η^2^ = 0.025	F(2,80) = 0.270, *p* = 0.764, η^2^ = 0.007	—
Steering Angle SD	A: Baseline	1.10 ± 1.14	1.23 ± 1.37	0.60 ± 0.33	2.29 ± 1.21	*F*(3,120) = 20.673, *p* < 0.001, η^2^ = 0.341
	B: Markings	0.85 ± 0.74	1.06 ± 1.43	0.58 ± 0.41	2.00 ± 0.97	F(3,120) = 19.503, *p* < 0.001, η^2^ = 0.328
	C: Markings+Horn	1.09 ± 1.07	1.04 ± 0.85	0.59 ± 0.37	2.26 ± 1.13	F(3,120) = 33.059, *p* < 0.001, η^2^ = 0.452
Cross-scenario statistics	—	*F*(2,80) = 1.238, *p* = 0.296, η^2^ = 0.030	F(2,80) = 0.433, *p* = 0.650, η^2^ = 0.011	F(2,80) = 0.009, *p* = 0.991, η^2^ < 0.001	F(2,80) = 2.322, *p* = 0.105, η^2^ = 0.055	—

Data are presented as Mean ± Standard Deviation (SD). P95 and P5 indicate the 95th and 5th percentiles, respectively. Longitudinal statistics represent repeated-measures ANOVA comparisons across the four spatial phases within each scenario, whereas cross-scenario statistics represent repeated-measures ANOVA comparisons across the three warning scenarios within each spatial phase. Significant omnibus scenario effects were followed by paired comparisons with Holm correction. η^2^ denotes partial eta squared. Bold values indicate statistically significant cross-scenario omnibus effects (*p* < 0.05). *p < 0.05, ***p < 0.001.

The mean speed reflects drivers’ macroscopic compliance with speed-limit targets. Significant spatial variation in mean speed was observed within all three warning scenarios (all *p* < 0.001; Table 1). Upon entering Phase IV, the mean speeds decreased to approximately 59–61 km/h, approaching the 60 km/h speed-management target. Cross-scenario comparisons showed no statistically significant differences in Phases I, II, and IV. However, a significant scenario effect was observed in Phase III, *F*(2, 80) = 3.381, *p* = 0.039, η^2^ = 0.078. Holm-adjusted paired comparisons further indicated that Scenario C produced a lower mean speed than Scenario A, t(40) = 2.572, adjusted *p* = 0.042, whereas the other pairwise comparisons were not significant. Overall, the warning modalities produced largely comparable macroscopic speed outcomes across most spatial phases, with a localized speed-control difference emerging in Phase III.

(2) Microscopic distribution characteristics of ultimate initial braking intensity.

The 95th percentile braking pedal force (P95) was used to characterize the upper range of braking intensity across the four spatial phases. Significant spatial variation was observed within all three scenarios (all *p* < 0.001; Table 1), with the highest P95 values occurring in Phase I (8.92–9.47%) and values decreasing to near zero in Phase II. In contrast, no significant cross-scenario differences were found in any of the four phases (all *p* > 0.05; Table 1). These results indicate that the addition of visual markings and directional auditory warnings did not produce a detectable increase in extreme braking intensity relative to the baseline condition.

(3) Spatial characteristics of longitudinal control smoothness.

Peak negative jerk (Jerk P5) was used to characterize microscopic longitudinal control smoothness. Significant spatial variation in Jerk P5 was observed within all three warning scenarios (all *p* < 0.001; Table 1). A significant overall scenario effect emerged in Phase I, *F*(2, 80) = 4.392, *p* = 0.016, η^2^ = 0.099, whereas no significant scenario effects were observed in Phases II–IV. In Phase I, Scenario A exhibited a more negative mean Jerk P5 (−1.10 m/s^3^) than Scenario B (−0.75 m/s^3^) and Scenario C (−0.78 m/s^3^), indicating descriptively smoother longitudinal control under the enhanced warning conditions. However, the pairwise differences did not remain statistically significant after Holm correction. These results suggest a potential early-stage smoothing effect of the enhanced warning configurations, while the pairwise evidence should be interpreted cautiously.

(4) Rigid geometric bottlenecks of lateral trajectory stability.

The standard deviation of steering wheel angle was used to characterize lateral control variability. Significant spatial variation was observed within all three warning scenarios (all *p* < 0.001; Table 1), with steering-angle variability reaching its highest level in Phase IV. However, no significant cross-scenario difference was observed in Phase IV, *F*(2, 80) = 2.322, *p* = 0.105, η^2^ = 0.055. This pattern suggests that the increase in lateral control variability near the work-zone boundary was more closely associated with the changing geometric environment than with the warning modality itself. Thus, although advance warning facilities may influence longitudinal speed-control behavior, their effects on lateral control appear limited when drivers approach the physical lane-constriction area.

#### 3.3.3 Spatiotemporal interaction and heterogeneity deconstruction based on LMM

To profoundly investigate whether the intervention efficacy of multimodal warning facilities exhibits spatiotemporal evolutionary characteristics as vehicles approach the work zone, this study introduced a “Warning Scenario × Spatial Phase” deep interaction term into the LMM framework. Previous macroscopic spatial evolution analyses have confirmed that warning facilities can effectively guide the traffic flow to complete the overall deceleration transition. However, the outbreak of rear-end conflicts upstream of work zones typically does not depend on the average cross-sectional speed of the traffic flow, but is rather directly triggered by sudden emergency evasions or microscopic control instability. Therefore, in the in-depth modeling stage aimed at isolating individual heterogeneity, this study proactively discarded conventional macroscopic speed indicators. Instead, it focused on the “maximum longitudinal deceleration,” which represents the rigid boundary of physical braking, and the “peak negative longitudinal jerk (Jerk P5),” a second-order dynamic indicator quantifying microscopic maneuvering smoothness, as the core analytical targets.

Multicollinearity diagnostics showed that the VIFs were 5.33 for the warning-scenario indicators and 4.50 for the spatial-phase indicators, while all Warning Scenario × Spatial Phase interaction terms had VIFs below 5. Under the more conservative reference value of 5, the warning-scenario indicators showed a slight elevation, whereas the spatial-phase indicators and interaction terms remained below this level. Accordingly, the diagnostics suggest modest collinearity associated with the scenario indicators, but no evidence of severe multicollinearity in the fixed-effect structure.

Model estimation and variance decomposition results (Table 2) reveal that the Intraclass Correlation Coefficients (ICC) for Jerk P5 and maximum longitudinal deceleration reached 0.192 and 0.157, respectively. This demonstrates that under the high-pressure speed control context upstream of the work zone, approximately 15.7 to 19.2% of the acceleration and deceleration fluctuations essentially stem from inherent differences in drivers’ innate pedal-operation habits and driving styles. By introducing participant-level random intercepts, the LMM successfully filtered out this unobserved subjective operational noise, thereby unbiasedly restoring the true physical intervention efficacy of the warning facilities.

**Table 2:** Model estimates of spatiotemporal intervention effects of work zone warnings stripping individual heterogeneity.

Variables and effects	Estimate	StdErr.	z	*p*	95% CI
Panel A: (Jerk P5)					
Fixed main effects					
Intercept: Scenario A, Phase I	−1.118	0.1	−11.137	<0.001	[−1.315, −0.922]
Scenario B	0.3	0.128	2.348	0.019	[0.050, 0.550]
Scenario C	0.312	0.128	2.442	0.015	[0.062, 0.563]
Phase II	0.723	0.128	5.657	<0.001	[0.472, 0.973]
Phase III	0.13	0.128	1.016	0.31	[−0.121, 0.380]
Phase IV	0.065	0.128	0.51	0.61	[−0.185, 0.316]
Interaction effects					
Scenario B × Phase II	−0.263	0.181	−1.458	0.145	[−0.618, 0.091]
Scenario C × Phase II	−0.361	0.181	−1.996	0.046	[−0.715, −0.007]
Scenario B × Phase III	−0.134	0.181	−0.741	0.458	[−0.488, 0.220]
Scenario C × Phase III	−0.224	0.181	−1.239	0.215	[−0.578, 0.130]
Scenario B × Phase IV	−0.275	0.181	−1.519	0.129	[−0.629, 0.080]
Scenario C × Phase IV	−0.187	0.181	−1.036	0.3	[−0.541, 0.167]
Random effects					
Individual variance	0.082	—	—	—	—
ICC	0.192	—	—	—	—
Panel B: (Max Deceleration)					
Fixed main effects					
Intercept: Scenario A, Phase I	−6.894	0.304	−22.64	<0.001	[−7.491, −6.297]
Scenario B	0.015	0.396	0.037	0.971	[−0.761, 0.790]
Scenario C	0.451	0.396	1.138	0.255	[−0.326, 1.227]
Phase II	6.021	0.396	15.215	<0.001	[5.245, 6.797]
Phase III	2.976	0.396	7.519	<0.001	[2.200, 3.751]
Phase IV	3.979	0.396	10.054	<0.001	[3.203, 4.754]
Interaction effects					
Scenario B × Phase II	0.335	0.56	0.598	0.55	[−0.762, 1.432]
Scenario C × Phase II	−0.136	0.56	−0.243	0.808	[−1.233, 0.961]
Scenario B × Phase III	−0.07	0.56	−0.125	0.901	[−1.167, 1.027]
Scenario C × Phase III	−0.037	0.56	−0.066	0.948	[−1.134, 1.060]
Scenario B × Phase IV	−0.455	0.56	−0.813	0.416	[−1.552, 0.642]
Scenario C × Phase IV	−0.649	0.56	−1.159	0.246	[−1.746, 0.448]
Random effects					
Individual variance	0.611	—	—	—	—
ICC	0.157	—	—	—	—

The reference level for the fixed effects is set to the observations of the baseline Standard Configuration Group (Scenario A) in the Initial Approach Zone (Phase I). To effectively filter out high-frequency noise induced by mechanical and environmental factors, the Jerk indicator extracts the 5th percentile extreme value (P5) of the negative longitudinal jerk within each spatial phase.

After strictly controlling for individual heterogeneity, the model’s fixed effects clearly unveiled an asymmetric intervention characteristic characterized by the coexistence of a “robust macroscopic braking baseline” and “reshaped microscopic deceleration processes.” Regarding the maximum longitudinal deceleration (Panel B), neither the fixed main effects nor the spatial interaction terms of any warning scenarios reached statistical significance (*p* > 0.05). This result provides solid quantitative evidence: when confronting the dual rigid compulsions of physical barricade blockages and mandatory speed limit signs, regardless of how the modalities of external warning facilities are upgraded, they cannot substantially alter the ultimate braking depth that drivers are forced to execute in extreme evasive situations to avoid collisions. The safety baseline of the vehicle’s physical braking exhibits remarkable robustness.

However, the safety-related value of the enhanced warning configurations is further reflected in the spatiotemporal evolution of microscopic longitudinal jerk (Panel A). In the Initial Approach Zone (Phase I), the fixed main-effect coefficients for Scenario B (continuous markings) and Scenario C (augmented visual–auditory warnings) were both significantly positive. Because peak negative jerk represents the lower tail of longitudinal jerk, these positive coefficients indicate that the magnitude of negative jerk was reduced by approximately 0.3 m/s^3^ relative to Scenario A. Previous naturalistic-driving studies have shown that longitudinal jerk is sensitive to abrupt and safety-critical braking behavior and can serve as a useful surrogate indicator of driving aggressiveness and safety-critical maneuvers (Bagdadi and Várhelyi, 2011; Feng et al., 2017). Therefore, the observed 0.3 m/s^3^ reduction should be interpreted as a relative improvement in longitudinal control smoothness rather than as a universal threshold for sufficient safety benefit. Furthermore, the Scenario × Phase IV interaction terms were not statistically significant, indicating that no statistically detectable change in the relative jerk effect was observed between Phase I and Phase IV. Nevertheless, the practical safety significance of this magnitude should be further validated using real-world safety outcomes or surrogate conflict measures.

### 3.4 Microscopic exposure rate assessment of speed limit compliance in continuous spatial segments

To systematically deconstruct the spatiotemporal intervention efficacy of different warning modalities on drivers’ continuous speed control behaviors, this study introduces the core indicator of “Trajectory Speeding Exposure Rate” (TSER). This metric is strictly defined as the ratio of speeding trajectory points to the total valid trajectory points within a specific spatial control zone, aiming to precisely quantify the cumulative spatiotemporal effects of microscopic speeding behaviors.

Figure 15 illustrates the spatiotemporal speeding exposure characteristics of the three warning scenarios across various control zones. In the mid-segment cruising zone with an 80 km/h speed limit (Zone 1), the speeding exposure rates for all three vehicle groups remained at exceedingly high levels (74.9–76.1%). This common phenomenon profoundly indicates that over extended straight sections lacking continuous physical control constraints, the “advance deceleration effect” elicited by the initial warning (Phase I) has significantly decayed and dissipated. Drivers universally slipped into a state of deep speed adaptation and vigilance relaxation. As vehicles entered the deceleration transition zone (Zone 2, 80 km/h limit), the intervention efficacies of different modalities began to exhibit preliminary divergence. The speeding exposure rate of the baseline Standard Configuration Group (Scenario A) climbed contrarily to 78.7%. Conversely, the exposure rates for Scenarios B and C, which incorporated continuous or composite interventions, steadily declined to 72.8 and 71.1%, respectively. This preliminarily confirms that external multimodal stimuli can effectively shatter drivers’ cruising inertia, successfully inducing a proactive and defensive early deceleration intent.

**Figure 15:**
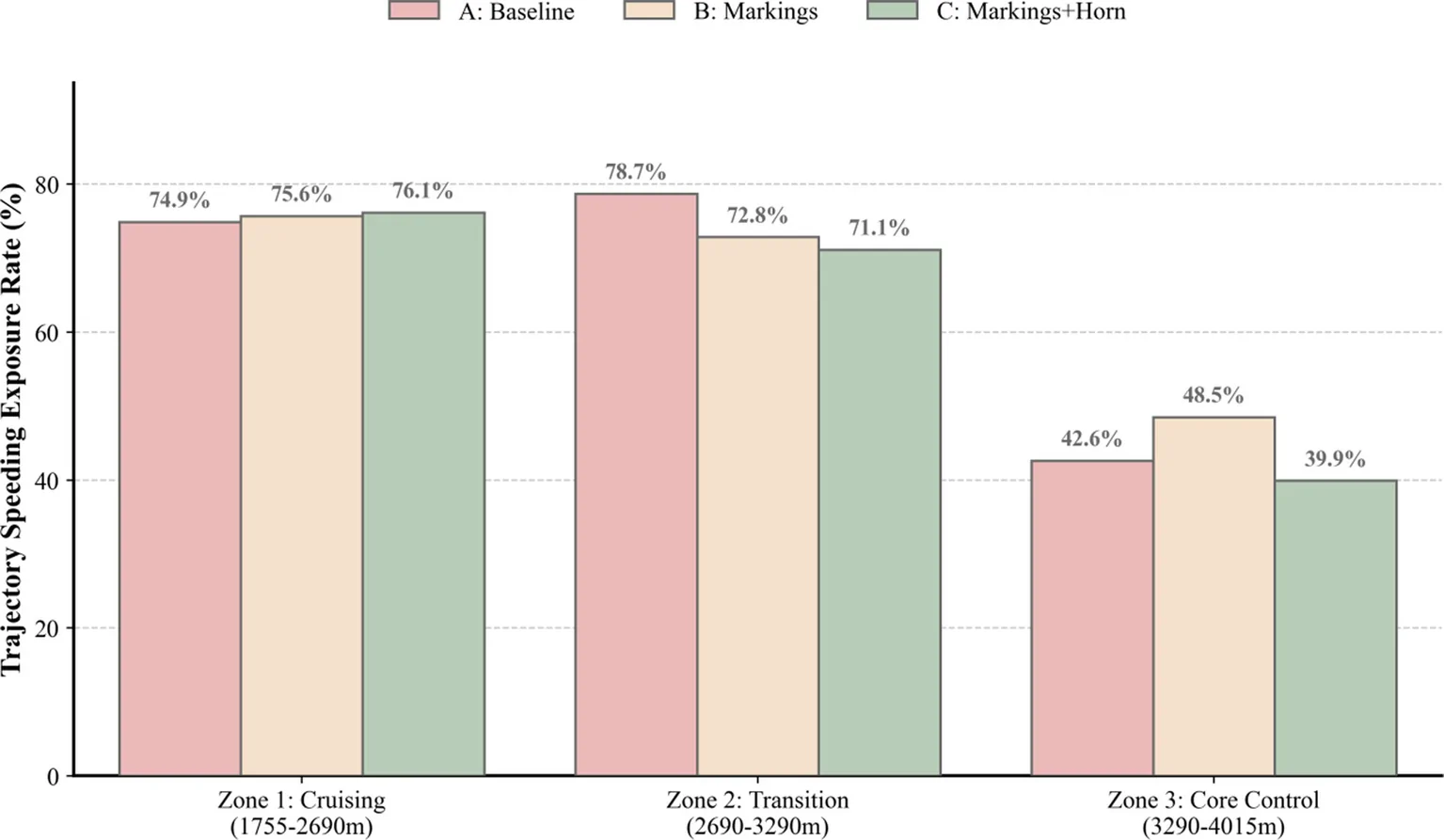
Comparison of trajectory speeding exposure rates across spatial control zones under different warning scenarios.

Upon entering the core control zone (Zone 3) with a 60 km/h speed limit, the speeding exposure characteristics demonstrated highly significant modal heterogeneity. The Augmented Warning Group (Scenario C) exhibited overwhelming speed control efficacy, with its speeding exposure rate plummeting to an overall low of 39.9%. However, it is highly noteworthy that the absolute speeding exposure rate of the single continuous marking group (Scenario B, 48.5%) was marginally higher than that of the unalerted baseline group (Scenario A, 42.6%). Integrating the previous spatial evolution findings regarding microscopic jerk, this seemingly “counter-intuitive” statistical phenomenon actually unravels two diametrically opposed underlying deceleration dynamics. The superficially lower speeding exposure rate of the baseline group (Scenario A) fundamentally stems from drivers executing panic hard braking due to a severe lack of psychological preparedness the moment they visually recognized the 60 km/h sign. While this precipitous speed drop mathematically truncated the duration of the speeding state, it physically triggered extremely high-risk longitudinal traffic shockwaves. In stark contrast, the continuous high-density visual guidance in Scenario B successfully transformed drivers’ aggressive braking into a much smoother, progressive deceleration process. Although the vehicle speed lingered slightly longer during the transition across the speed limit threshold (manifesting visually as a marginally higher speeding exposure rate), it fundamentally and entirely averted malignant longitudinal oscillations and secondary rear-end collision risks.

Synthesizing the microscopic dynamic and macroscopic compliance results, the differences between Scenarios B and C should primarily be interpreted in relation to the additional auditory component, because the two scenarios employed identical visual markings. Compared with Scenario B, Scenario C exhibited a lower speeding exposure rate in the final control zone, suggesting that the additional auditory prompts provided complementary reinforcement to the existing visual guidance during the terminal approach. This cross-modal reinforcement may have helped maintain the effectiveness of speed-control information as drivers approached the work-zone boundary, rather than reflecting visual fatigue. Overall, the visual–auditory warning configuration in Scenario C achieved a more favorable balance between microscopic maneuvering smoothness and macroscopic speed-limit compliance.

## 4 Discussion

Based on the driving simulator experiment, this study examined how different warning configurations influence drivers’ longitudinal speed-control behavior when approaching highway work zones. The results show that the effects of warning facilities are manifested differently at the macroscopic and microscopic levels. Although the three scenarios produced broadly comparable terminal deceleration outcomes, differences emerged in the evolution of the deceleration process and in specific spatial phases. These findings suggest that evaluating work-zone warning effectiveness solely on the basis of final speed or maximum deceleration may overlook important changes in how drivers regulate speed throughout the approach process.

Existing studies on work-zone safety have commonly used speeding behavior and speed-control performance as primary indicators. Previous research has shown that speeding may persist upstream of work zones and that drivers continuously adjust their behavior as they approach traffic-control facilities (Thapa et al., 2024). Consistent with these observations, participants in all three scenarios completed substantial deceleration before entering the core work-zone area. The LMM results further showed that maximum longitudinal deceleration did not differ significantly across warning scenarios after accounting for between-driver heterogeneity. This indicates that the final braking depth was strongly constrained by the mandatory speed limit and the physical configuration of the work zone. At the same time, the phase-based analysis revealed a localized scenario effect on mean speed in Phase III, where Scenario C produced a lower mean speed than Scenario A. This suggests that warning modality may influence the timing and spatial progression of deceleration even when the final speed-control outcome remains broadly similar.

This convergence in the terminal deceleration outcome is consistent with the behavioral-regulation perspective of Risk Homeostasis Theory, which proposes that drivers adjust their behavior in response to perceived changes in risk while tending to maintain an acceptable level of overall risk (Wilde, 1982). Similar compensatory responses have also been reported in studies of Advanced Driver Assistance Systems, where external warnings can alter short-term driving behavior without necessarily producing a persistent shift toward more conservative driving (Kim et al., 2022; Sullivan et al., 2016; Weaver et al., 2022). In the present work-zone context, the physical speed-limit requirement and lane-control environment therefore appear to impose a common terminal constraint on drivers’ deceleration behavior, while the warning configurations mainly affect how that deceleration is distributed over space and time.

More pronounced differences were observed in microscopic longitudinal control. In Phase I, the LMM showed significantly positive fixed effects for Scenarios B and C on Jerk P5 relative to Scenario A, corresponding to an approximately 0.3 m/s^3^ reduction in the magnitude of negative jerk. Because smaller negative jerk magnitudes indicate smoother longitudinal control, this result suggests that enhanced warning configurations can reduce the abruptness of the early deceleration process. However, this magnitude should be interpreted as a relative improvement in control smoothness rather than as a universal threshold for safety benefit. The non-significant Scenario × Phase IV interaction terms further indicate that no statistically detectable change in this relative effect was observed between the initial and terminal phases, rather than proving that the effect remained unchanged throughout the entire approach. These findings are consistent with previous studies showing that longitudinal jerk is sensitive to abrupt or safety-critical braking behavior and can provide useful information on driving smoothness and risk-related maneuvering.

From a cognitive perspective, the observed smoothing effect may be related to the way in which advance warning information is processed. Work-zone environments typically impose increasing visual and cognitive demands as drivers approach dense traffic-control facilities, including speed-limit signs, channelizing devices, lane markings, and work-zone boundaries (Tang and Adebisi, 2022; Yang et al., 2023). Under such conditions, drivers relying primarily on visual information may face greater competition for perceptual resources. The addition of auditory information provides a complementary channel through which risk information can be conveyed, which is consistent with Multiple Resource Theory (Wickens, 2008). Previous studies have similarly shown that combined visual and auditory warnings can improve drivers’ responses to safety-critical information (Biondi et al., 2017). The present results therefore support the interpretation that multimodal warnings may influence the organization of the deceleration process by supplementing visual guidance rather than simply producing stronger braking responses.

The effectiveness of auditory information also appeared to vary with spatial context. In the far-field cruising sections, isolated voice prompts produced relatively limited behavioral changes, whereas clearer differences emerged as vehicles approached the downstream control area. This spatial dependence suggests that the effectiveness of auditory warnings is not uniform along the work-zone approach. When visual information density increases close to the work-zone boundary, auditory cues may provide additional support for hazard recognition and speed regulation. In contrast, isolated auditory prompts delivered in relatively low-demand upstream sections may have weaker effects because drivers have more time and fewer immediate constraints. These findings support a spatially differentiated warning strategy rather than the uniform deployment of the same warning modality throughout the entire approach zone.

The results also highlight the value of incorporating process-based indicators into work-zone safety evaluation. Traditional cross-sectional measures such as mean speed and terminal speed compliance provide important information on whether drivers ultimately meet the required speed target, but they cannot fully characterize the dynamics of the deceleration process. Continuous trajectory-based measures, including longitudinal jerk and speeding exposure, can complement conventional speed indicators by capturing abrupt control adjustments, temporal instability, and the persistence of speeding behavior along the approach path. Accordingly, work-zone warning evaluation may benefit from combining macroscopic compliance indicators with microscopic process-oriented measures rather than relying on either type of measure alone.

The findings should nevertheless be interpreted in light of several limitations. First, the experiment was conducted in a driving simulator under a single-vehicle free-flow condition, and interactions with surrounding vehicles, car-following behavior, and traffic-density effects were not represented. Second, the experimental scenarios were derived from one work-zone configuration and a specific set of visual and auditory warning layouts, which may limit direct generalization to other geometric conditions, traffic environments, and warning designs. Third, the present study relied primarily on vehicle-dynamic and trajectory-based measures; although these indicators provide useful evidence of control smoothness and speed-regulation behavior, they do not directly measure crash occurrence or drivers’ internal cognitive states. Therefore, the results are best interpreted as evidence of behavioral and operational effects rather than direct proof of crash-risk reduction. Future studies could validate these findings using naturalistic or field driving data under different traffic-demand levels, incorporate surrounding-vehicle interactions and surrogate conflict measures, and further integrate eye-tracking data to examine the attentional mechanisms underlying the observed behavioral responses.

Overall, the findings indicate that enhanced work-zone warning configurations influence not only whether drivers achieve the required speed but also when and how they decelerate. Their principal contribution appears to lie in reshaping the spatial and temporal organization of the deceleration process, while the final braking magnitude remains largely constrained by the physical and regulatory characteristics of the work zone. This distinction provides a more nuanced basis for designing spatially differentiated warning systems and for evaluating their effectiveness using both outcome-based and process-based indicators.

## 5 Conclusion

Based on high-fidelity driving simulator experiments and continuous trajectory data, this study systematically evaluated the spatiotemporal intervention efficacy of multimodal warning facilities upstream of highway work zones from the dual perspectives of macroscopic speed control outcomes and microscopic dynamic processes. The primary research conclusions are as follows:

(1) When confronting the rigid physical barricades and a definitive speed limit target (60 km/h) of the work zone, neither the total cross-sectional deceleration magnitude nor the peak maximum deceleration exhibited significant inter-group differences across the three scenarios. Utilizing a Linear Mixed-Effects Model (LMM) to strictly strip away the data interference caused by inherent individual driving habits further corroborated this macroscopic homogenization characteristic. This indicates that the physical environment of the work zone constitutes an exceedingly strong physical constraint, and warning facilities cannot substantially alter the absolute baseline of drivers’ ultimate braking depth.
The core safety efficacy of the warning facilities is not reflected in a further reduction of the macroscopic absolute vehicle speed, but rather in reshaping the microscopic process of continuous vehicle deceleration. The absence of advance warnings easily leads drivers to execute panic hard braking the moment they visually recognize the signs, triggering high-risk longitudinal traffic shockwaves. In contrast, by releasing risk information in advance and prolonging drivers’ reaction times, multimodal continuous warnings globally softened hard braking behaviors from the initial intervention phase, significantly reducing the peak negative jerk and transforming the deceleration process into a much smoother and safer longitudinal trajectory.
The intervention efficacy of the warning modalities exhibits significant spatial variance. In the far-field extended straight cruising zone, isolated voice prompts are easily adapted to and ignored by drivers, demonstrating a clear decay effect. However, in the high-visual-workload bottleneck section approaching the work zone frontier, relying solely on continuous visual markings tends to prolong the deceleration transition period and elevate the speeding exposure rate. At this critical juncture, the introduction of auditory information achieves effective cross-modal visual–auditory reinforcement, successfully overcoming drivers’ operational inertia and vigilance decay, and drastically minimizing the spatiotemporal speeding exposure rate in the ultimate approach zone (to 39.9%).
Relying solely on discrete cross-sectional speed indicators is insufficient to comprehensively characterize the true safety hazards of work zones. Future proactive work zone safety evaluation frameworks should widely incorporate continuous trajectory-level Surrogate Safety Measures (SSMs), such as Jerk and the spatiotemporal speeding exposure rate, to accurately capture potential rear-end collision risks at the microscopic dynamic level.

Based on these findings, a spatially differentiated strategy is recommended for the design of upstream work-zone warnings. Specifically, composite warning facilities combining continuous visual markings and directional voice alerts could be preferentially deployed in high-risk bottleneck areas immediately upstream of the work-zone boundary to improve speed-limit compliance while maintaining longitudinal control smoothness. These findings provide a quantitative basis for optimizing the spatial deployment of multimodal warning facilities in highway work zones.

## Data Availability

The original contributions presented in the study are included in the article/supplementary material, further inquiries can be directed to the corresponding author.
